# A Versatile Strategy to Reduce UGA-Selenocysteine Recoding Efficiency of the Ribosome Using CRISPR-Cas9-Viral-Like-Particles Targeting Selenocysteine-tRNA^[Ser]Sec^ Gene

**DOI:** 10.3390/cells8060574

**Published:** 2019-06-11

**Authors:** Caroline Vindry, Olivia Guillin, Philippe E. Mangeot, Théophile Ohlmann, Laurent Chavatte

**Affiliations:** 1 Centre International de Recherche en Infectiologie (CIRI), 69007 Lyon, France; caroline.vindry@ens-lyon.fr (C.V.); olivia.guillin@ens-lyon.fr (O.G.); philippe.mangeot@ens-lyon.fr (P.E.M.); 2 Institut National de la Santé et de la Recherche Médicale (INSERM) Unité U1111, 69007 Lyon, France; 3 Ecole Normale Supérieure de Lyon, 69342 Lyon, France; 4 Université Claude Bernard Lyon 1 (UCBL1), 69622 Lyon, France; 5 Unité Mixte de Recherche 5308 (UMR5308), Centre National de la Recherche Scientifique (CNRS), 69007 Lyon, France

**Keywords:** selenium, selenocysteine, selenoprotein, SECIS, Sec-tRNA^[Ser]Sec^, UGA-recoding, CRISPR-Cas9, viral-like particles, nanoblades

## Abstract

The translation of selenoprotein mRNAs involves a non-canonical ribosomal event in which an in-frame UGA is recoded as a selenocysteine (Sec) codon instead of being read as a stop codon. The recoding machinery is centered around two dedicated RNA components: The selenocysteine insertion sequence (SECIS) located in the 3′ UTR of the mRNA and the selenocysteine-tRNA (Sec-tRNA^[Ser]Sec^). This translational UGA-selenocysteine recoding event by the ribosome is a limiting stage of selenoprotein expression. Its efficiency is controlled by the SECIS, the Sec-tRNA^[Ser]Sec^ and their interacting protein partners. In the present work, we used a recently developed CRISPR strategy based on murine leukemia virus-like particles (VLPs) loaded with Cas9-sgRNA ribonucleoproteins to inactivate the Sec-tRNA^[Ser]Sec^ gene in human cell lines. We showed that these CRISPR-Cas9-VLPs were able to induce efficient genome-editing in Hek293, HepG2, HaCaT, HAP1, HeLa, and LNCaP cell lines and this caused a robust reduction of selenoprotein expression. The alteration of selenoprotein expression was the direct consequence of lower levels of Sec-tRNA^[Ser]Sec^ and thus a decrease in translational recoding efficiency of the ribosome. This novel strategy opens many possibilities to study the impact of selenoprotein deficiency in hard-to-transfect cells, since these CRISPR-Cas9-VLPs have a wide tropism.

## 1. Introduction

**Figure 1:**
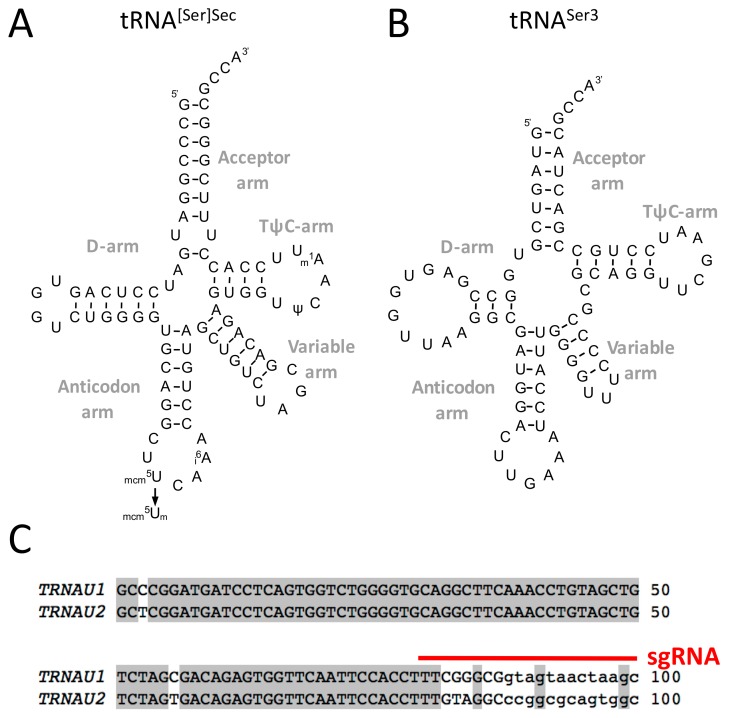
The tRNA^[Ser]Sec^, two-dimensional structure, modification, and comparison to tRNA^Ser3^ and its pseudogene. Two-dimensional structure representation of tRNA^[Ser]Sec^ encoded by *TRNAU1* gene (**A**) and tRNA^Ser3^ (**B**). (**C**) Sequence alignment of human *TRNAU1* and *TRNAU2* genes, located in chromosome 19 and 22, respectively. The common nucleotides are represented in grey, otherwise in white. The region targeted by the sgRNA is shown with a red bar.

Selenocysteine (Sec) is the 21st proteinogenic amino acid and it was the first addition to the genetic code deciphered in the 1960s. Selenocysteine, a cysteine analogue with selenium replacing sulphur, is co-translationally inserted in the protein sequence via an unusual translational mechanism that consists of the recoding of an UGA codon which is normally read as a stop codon for other cellular mRNAs [1,2,3,4,5,6,7]. In human, 25 selenoprotein genes have been identified [8], in which the termination signal is often one of the two other stop codons, namely UAA or UAG. This UGA/Sec recoding process is possible due to two dedicated RNA components and their interacting partners. First, the selenocysteine insertion sequence (SECIS) is a stem-loop structure of approximately 100 nucleotides that is located in the 3′ UTR of all selenoprotein mRNAs [9,10]. The SECIS is necessary and sufficient to drive the efficient recoding of an in-frame UGA codon. This feature has been particularly convenient when performing a structure–function analysis of the SECIS element in heterologous gene systems, such as luciferase reporter constructs containing the SECIS in the 3′ UTR [9,11,12,13,14,15]. The SECIS element serves as a dynamic platform to recruit the SECIS binding protein 2 (SECISBP2) and other Sec dedicated recoding factors [14,16,17,18,19,20]. The second key RNA component of Sec insertion machinery is the Sec-tRNA^[Ser]Sec^ (Figure 1A) which associates with a selenocysteine-specific elongation factor (EFSec) [17,18].

One Sec-tRNA^[Ser]Sec^ gene is present in the human genome in chromosome 19 (*TRU-TCA1-1 or TRNAU1*) [21]. A pseudogene is also found in chromosome 22 (*TRU-TCA2-1* or *TRNAU2*) and contains mutations leading to an inactive acceptor arm (see Figure 1C). In addition, *TRNAU2* does not seem to be transcribed by RNA polymerase (Pol) III [21]. Strikingly, the Sec-tRNA^[Ser]Sec^ is the only known tRNA that governs by itself the expression of an entire group of proteins, the selenoproteome, which is composed by 25 selenoprotein genes [2,22]. Therefore, in contrast to other cellular tRNAs, the inactivation of the tRNA^[Ser]Sec^ could be achieved by only one gene disruption. In mice, its gene inactivation (*Trsp∆*) leads to embryonic lethality at stage E6.5, indicating the essentiality of at least one selenoprotein in mammalian development [23]. To study the role of selenoproteins in various tissues, the inactivation of selenocysteine insertion is achieved by crossing mice carrying a conditional allele of Sec-tRNA^[Ser]Sec^ (*Trsp^fl/fl^*) with tissue-specific *Cre* strains [24]. To date, the removal of mouse Trsp was reported in mammary glands, liver, kidney, heart, thyroid, skeletal muscle, prostate, skin, endothelial cells, T-cells, macrophages, osteo-chondroprogenitors, and neurons with different phenotypes (as reviewed in Reference [24]).

**Figure 2:**
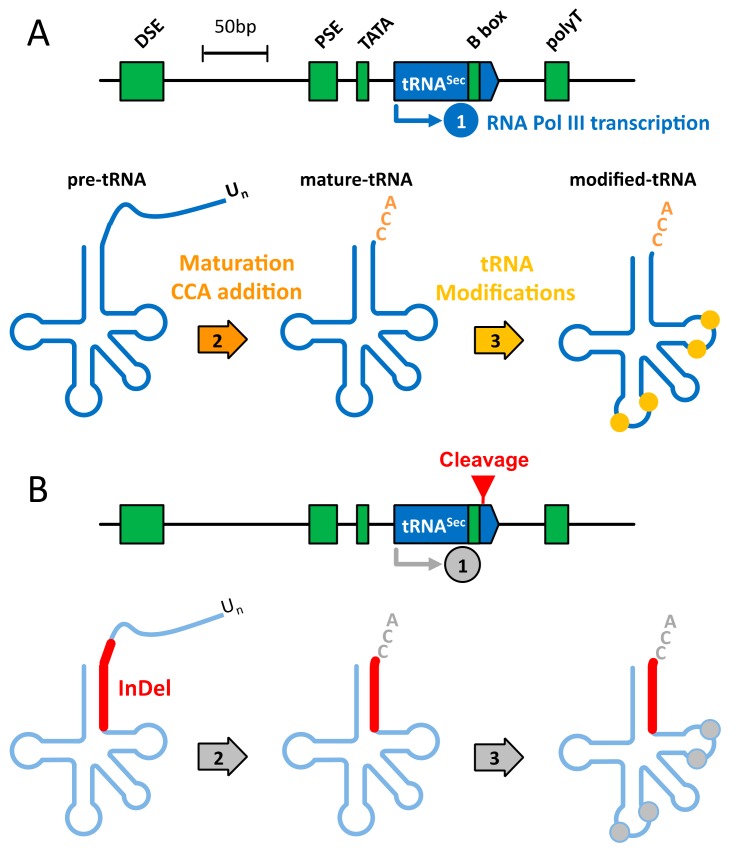
Design of a clustered regularly interspaced short palindromic repeats (CRISPR) strategy that disrupts Sec-tRNA^[Ser]Sec^ expression and function. (**A**) Schematic representation of the transcription by RNA PolIII (1), maturation (2), and modification (3) stages of wild type tRNA^[Ser]Sec^ from the *TRNAU1* gene. (**B**) Illustration of the various possible consequences of our CRISPR strategy. In response to the dsDNA break caused by Cas9-sgRNA in Table S1. Nucleotide insertion or deletion (Indel) can occur at the cutting site due to imperfect repair by NHEJ. This Indel can impact the tRNA^[Ser]Sec^ expression at three levels: (1) The efficiency of transcription by altering the B box and/or the termination site, (2) the removal of the 3’-trailer and the CCA-addition at the 3′-end of the tRNA by altering the recognition of the tRNA by the respective enzymes (tRNA endonuclease and tRNA nucleotidyltransferase), and (3) the modifications of the tRNA. In any case, the action of Cas9-sgRNA at this site leads to impaired and/or reduced levels of tRNA^[Ser]Sec^. The tRNA sequence and the important regions for the transcription are represented in blue and green, respectively. DSE, distal sequence element; PSE, proximal sequence element; TATA, TATA box; polyT, transcription stop site.

The Sec-tRNA^[Ser]Sec^ harbors many different features in terms of size, structure, transcription, modification, aminoacylation, and transport [1,2,3,22] that make it unique in comparison with the other cytoplasmic tRNAs. First, with 96 nucleotides in length, it is by far the largest tRNA in eukaryotes. Then, the relative ratio between the acceptor arm size (expressed in base pairs (bp)) versus T**ψ**C arm is distinct from canonical tRNAs. The Sec-tRNA^[Ser]Sec^ folds in a 9/4 secondary structure instead of 7/5 in other cellular tRNAs (see Figure 1A,B). In addition, the variable arm is particularly large with 16 nucleotides folded in a stem loop. These features not only prevent it from interacting with the elongation factor EF-1A but they are also used to specifically interact with EFSec. The transcription of *TRNAU1* gene in pre-tRNA^[Ser]Sec^ by RNA Pol III is also singular. Instead of having the two intragenic Box A and B sequences, the tRNA^[Ser]Sec^ gene has three upstream promoters: a TATA box, a proximal sequence element (PSE) and a distal sequence element (DSE); and one intragenic Box B as illustrated in Figure 2A. Interestingly, this unusual transcription causes a 5′ leaderless pre-tRNA^[Ser]Sec^ with only the 3′-end to be processed into a mature tRNA. In terms of post-transcriptional modifications, only four modified bases are found in Sec-tRNA^[Ser]Sec^ which is in the lower range for tRNAs (Figure 2A). Methyladenosine (m^1^A) at position 58 and pseudouridine (**ψ**) at position 55 are both important for the tRNA folding [25,26]. In the anticodon loop, one finds two other modified bases that are critical for UGA recoding, namely the 5-methoxycarbonylmethyl-uridine (mcm^5^U) at position 34 and N^6^-isopentenyladenosine (i^6^A) at position 37. Interestingly the mcm^5^U34 base, which is in the wobble position in tRNA^[Ser]Sec^ can be further methylated into 5-methoxycarbonylmethyluridine-2′-*O*-methylribose (mcm^5^Um). Since this latter methylation reaction is not complete, two isoforms (mcm^5^U34 *vs* mcm^5^Um34) co-exist in the cytoplasm, the methylated form being stimulated by selenium supplementation both in cell and animal models [27,28]. Interestingly, mouse models missing mcm^5^Um34 are unable to synthesize several selenoproteins including Gpx1, SelenoW, and Msrb1 [29]. In contrast to other proteogenic amino acids, selenocysteine is not charged as such on its dedicated tRNA but it is instead synthesized onto the tRNA from the amino acid serine, its oxygen analog, and hydrogen selenide (HSe^−^) as the selenium donor. Therefore, the aminoacylation of Sec-tRNA^[Ser]Sec^ involves four enzymes rather than only the amino acid-tRNA synthetase (aaRS) for other tRNAs [1,2,3,22]. Namely, the seryl-tRNA synthetase (SerRS), the phosphoseryl-tRNA kinase (PSKT), Sec synthase (SepSecS), and selenophosphate 2 synthetase (Sephs2) are required for the charging of a serine amino acid which is further transformed into a selenocysteine. Finally, concerning its transport, EFSec has evolved from EF1A to form a complex with the charged Sec-tRNA^[Ser]Sec^. In one of the current models for selenocysteine insertion, the EFSec/GTP/Sec-tRNA^[Ser]Sec^ ternary complex is recruited by the SECISBPP2/SECIS complex in the 3′ UTR of selenoprotein mRNAs to deliver the tRNA to the ribosome when an UGA codon occupies the A site [1,3]. Nevertheless, the sequence of events leading to selenocysteine insertion awaits further analysis, notably at the level of a three-dimensional structure and mRNP complexes characterization.

Among the genome editing tools, the clustered regularly interspaced short palindromic repeats (CRISPR) have revolutionized the research in life science by allowing simple and versatile gene engineering [30]. CRISPR is a two component system composed of the bacterial derived nuclease CRISPR-associated protein 9 (Cas9) associated with the single-guide RNA (sgRNA) designed to target the complementary DNA sequence in genomic DNA (gDNA). This complex induces a double strand DNA (dsDNA) break at the specific locus, which is then repaired by the cell using the non-homologous end-joining (NHEJ) machinery. In most cases, the resulting gDNA is different from the original sequence with several nucleotides insertion or deletion (Indel) at the cutting site. CRISPR strategies result in either knock-out or knock-down depending on the experimental design [31]. Our lab has recently developed a protein-delivery vector, named Nanoblades, which is a viral- like particle (VLP) based on the murine leukemia virus (MLV) which allows the delivery of Cas9-sgRNA ribonucleoproteins (RNPs) to various cells [32]. These Nanoblades are devoid of genetic material and lead to a rapid, transient, and efficient action of the Cas9-sgRNA RNPs on the gDNA, even in cells that are technically difficult to transfect.

Here, we have engineered a novel method allowing a robust reduction of selenoproteins in various cell lines by targeting the 3′-side of the acceptor arm of Sec-tRNA^[Ser]Sec^ using CRISPR-Cas9-VLPs. We showed an efficient Indel score at the specified gDNA locus in six different cell lines, namely Hek293, HepG2, HaCaT, HAP1, HeLa, and LNCaP. We observed an efficient down-regulation of the Sec-tRNA^[Ser]Sec^ levels in response to genomic alteration which provokes a decrease in selenoprotein expression. In summary, we report a novel experimental strategy to study selenoprotein deficient cell lines without affecting selenium levels. As such, it was designed to knock-down Sec-tRNA^[Ser]Sec^ expression to a level that was sufficient to drastically down-regulate selenoprotein synthesis without affecting cell viability.

## 2. Materials and Methods

This manuscript adopts the new systematic nomenclature of selenoprotein names [33].

### 2.1. Cell Culture

Hek293, HeLa (Life Technologies, Carlsbad, CA, USA, Cat.# R75007 and R71407), HAP1 (Horizon Discovery, Cambridge, UK, Cat.# C631), HaCaT (CLS Cell Lines Service GmbH, Eppelheim, Germany, Cat.# 300493 ), LNCaP (ATCC, Manassas, VA, USA, Cat.# CRL-1740), Gesicle producer 293T (Clontech, Mountain View, CA, USA, Cat.# 632617) were grown and maintained in 75 cm^2^ plates in Dulbecco’s Modified Eagle Medium (D-MEM). HepG2 (ATCC, Cat.# HB-8065) were similarly cultured in Eagle’s Minimum Essential Medium (MEM) medium. Media were supplemented with 10% fetal calf serum, 100 µg/mL streptomycin, 100 UI/mL penicillin, 2 mM L-glutamine (Thermo Fisher Scientific, Waltham, MA, USA). Cells were cultivated at 37 °C in humidified atmosphere containing 5% of CO_2_.

### 2.2. Production of Viral-Like-Particles

sgRNA coding sequence was cloned into pBlade plasmid as previously described [32]. One microliter of each primer (10 pmol/µL) 5’-caccgCTTAGTTACTACCGCCCGAA-3’ (*TRNAU1*) and 5’-aaacTTCGGGCGGTAGTAACTAAGc-3’ (*EMX-1*) were hybridized into 50 µl of 1X New England Biolabs Buffer 2. The mixture was heated at 94 °C for 2 min and slowly decreased to 40 °C. One microliter of the hybridized mix was used in a ligation reaction (New England Biolabs, Ipswitch, MA, USA) containing 200ng of pBlade digested by BsmBI and 1 µL of T4DNA-Ligase. This operation resulted in the insertion of the gRNA sequence upstream of the pBlade U6-promoter to drive tRNA-gRNA expression in transfected cells. Plasmid transformation was performed into 5-alpha Competent *E.coli* (New England Biolabs) according to the manufacturer’s instructions.

VLPs (Nanoblades) were produced in gesicles producer 293T cells according to Reference [32]. In summary, cells were platted at 3.5 × 10^6^ cells/10 cm plate 24 h before transfection with JetPrime reagent (Polyplus, Illkirch-Graffenstaden, France). Plasmids encoding gRNA (4.5 μg), Gag-PolMLV (3.3 μg), GagMLV-CAS9 fusion (2.0 μg), Baboon Endogenous retrovirus Rless glycoprotein (BaEVRless) (0.7 μg), and Vesicular stomatitis virus glycoprotein (VSV-G) (0.5 μg) were co-transfected. Supernatants were collected, filtered and concentrated by ultracentrifugation at 4 °C (1 h 30 min, 69,000× *g*). The concentrated particles from one 10 cm plate were resuspended in 100 µL PBS, aliquoted, and stored at −80 °C for several months or at +4 °C for several weeks.

To quantify the amount of Cas9 packaged into particles, VLPs or recombinant Cas9 (New England Biolabs) were diluted in Reporter Lysis 0,5× Buffer (Promega, Madison, WI, USA) and serial dilutions were spotted onto a nitrocellulose membrane. After incubation with a blocking buffer (nonfat milk 5% w/v in Tris buffer saline with 0.1% Tween20 (TBST)), the membrane was incubated with a Cas9 antibody coupled with HRP (7A9-3A3 clone, Cell signaling Technology, Danvers, MA, USA). Cas9 spots were revealed with ECL Select reagent by the Chemidoc touch imaging system (Biorad, Hercules, CA, USA) and analyzed with ImageLab Software (Biorad, Version 6.0.1).

### 2.3. Transduction Procedure

Cells were platted in a minimal volume to optimize cell/particles interactions for at least 2 h before supplementation with fresh medium. The indicated amount of VLP preparation was added to the medium for 2 × 10^5^ adherent cells. Cells were harvested after 4 days for genomic DNA extraction or grown for another 4 days in medium supplemented or not with 100 nM sodium selenite for protein and RNA extractions. For the rescue experiment, cells were transfected with the *TRNAU1* containing plasmid using TurboFect Transfection Reagent (Thermo Fisher Scientific) according to the manufacturer’s instructions 4 days after transduction.

### 2.4. Genomic DNA Extraction and Analysis

Genomic DNA was extracted from VLP-treated cells using the Nucleospin gDNA extraction kit (Macherey–Nagel). One hundred fifty nanograms of genomic DNA was then used for polymerase chain reaction (PCR) amplification with primers: Fw: 5′-CAGGGCTGTCACCCACCGCTGCGTCCTC-3′ Rev: 5′-GTCAACCATCTCACACCTTTCCAAAGG-3′.

For the T7 endonuclease1 mismatch assay, PCR products were diluted twice and complemented with Buffer 2 (New England Biolabs). Diluted PCR amplicons were then denatured at 95 °C and cooled down to 20 °C with a 0.1 °C/s ramp. Heteroduplexes were incubated for 15 min at 37 °C with T7 endonuclease 1 (New England Biolabs). Samples were run on a 2.5% agarose gel stained with ethidium bromide. Quantifications were performed with ImageLab software (Bio Rad).

To perform the Tracking of Indels by DEcomposition (TIDE), we sequenced the PCR products with the rev primer (5′-GTCAACCATCTCACACCTTTCCAAAGG-3′) and uploaded the .ab1 files to the online software (https://tide.deskgen.com) [34]. The parameters used for the TIDE analysis were as follows: Left boundary, 334; decomposition window (bp), 333–446; Indel size range, 2–10.

To analyze the mutants generated by Cas9, the region encompassing the tRNA gene was amplified by PCR from the gDNA extracted from cells treated with tRNA VLPs using the forward (5′-GGGGCCAGGGTGAATCAGACTC-3′) and reverse primers (5′-TCCGGAGGGGGAAATAAGTAACG-3′) with the Gotaq polymerase (Thermo Fisher Scientific). The PCR products from Hek293 and HAP1 gDNA were cloned into a pCRII TA cloning vector according to manufacturer’s instructions. The plasmid DNAs from a total of 57 and 58 colonies were extracted and sequenced for Hek293 and HAP1 cells, respectively.

### 2.5. Total RNA Extraction and Analysis by Northern Blot or RT-qPCR

Total RNAs were purified with Tri Reagent^®^ (Molecular Research Center, Cincinnati, OH, USA) according to the manufacturer’s instructions and resuspended in water. Fifteen micrograms of purified RNAs were size-fractioned on a TBE 1×, Urea (8M) polyacrylamide (15%) gel and electroblotted onto a Hybond-N nylon membrane (GE Healthcare, Chicago, IL, USA). The blots were hybridized overnight at 58 °C with 5′ terminally IRdye-labeled sequence-specific oligonucleotides (Integrated DNA technologies, Leuven, Belgium): tRNA-Sec 5′-(IRD800) CCACTGAGGATCATCCGGGC-3′ and tRNA-Ser 5′-(5IRD700) CGTAGTCGGCAGGATTCGAA-3′ [35] in PerfectHyb^TM^ Plus Hybridization Buffer (Sigma–Aldrich, Saint-Louis, MO, USA). Membranes were washed with 1× SSC/0.1% SDS buffer twice at 37 °C, and then once at room temperature. The infrared signal from the membrane was detected with an Odyssey imaging system CLx (LI-COR Biosciences, Lincoln, NE, USA). Quantifications were performed using ImageStudioLite software (Version 5.2.5, LI-COR Biosciences).

RNAs were reverse transcribed using qScript cDNA Synthesis kit (Quanta Bio, Beverly, MA, USA) according to the manufacturer’s instructions. Real time PCR was performed in triplicate using FastStart Universal SYBR^®^ Green master (Roche Applied Science, Penzberg, Germany) on a StepOne Real-Time PCR System (Applied Biosystems, Foster City, CA, USA). Primers used are described in Reference [36] and listed in Table S1. Serial dilutions of a cDNA mixture were used to create a standard curve and determine the efficiency of the amplification for each couple of primers.

### 2.6. Protein Extraction and Analysis by Western Blot

Cellular protein extracts were harvested, from 6-well plates, with 150 µL passive lysis buffer containing 25 mM Tris phosphate, pH 7.8, 2 mM DTT, 2 mM EDTA, 1% Triton X-100 and 10% glycerol. Next, protein concentrations were measured using the DC kit protein assay kit (Biorad) in microplate assays.

Equal protein amounts (30 µg) were separated in Bis-Tris NuPAGE Novex Midi Gels and transferred onto nitrocellulose membranes using iBlot^®^ DRy blotting System (Themo Fisher Scientific). Membranes were probed with indicated primary antibodies and HRP-conjugated anti-rabbit or anti-mouse secondary antibodies as described. Antibodies were purchased from Abcam (Cambridge, UK) (Gpx1, #ab108429; Gpx4, #ab125066; TxnRD1, #ab124954); Sigma-Aldrich (Actin, #A1978, HRP-conjugated goat anti-rabbit IgG, #A6154, HRP-conjugated goat anti-mouse IgG, #A9044). The chemiluminescence signal was detected using an ECL Select detection kit (GE Healthcare) in the Chemidoc Imager (Bio Rad). Data quantifications were performed with ImageLab software (Bio Rad).

## 3. Results

### 3.1. Design of a CRISPR-Cas9-Viral-Like-Particles Targeting the Sec-tRNA^[Ser]Sec^ (TRNAU1) Gene

The Sec-tRNA^[Ser]Sec^ gene is essential in mice and conditional knock-out mice have a peculiar phenotype in all the tissues studied [2,23]. Therefore, a complete knock-out of this gene in human cell lines would probably result in lethality or the strong perturbation of cell growth. Thus, we aimed at altering the Sec-tRNA^[Ser]Sec^ gene to decrease overall selenoprotein expression without affecting cell viability. As illustrated in Figure 2B, we designed a CRISPR strategy targeting the 3′- side of the acceptor arm of Sec-tRNA^[Ser]Sec^. After cutting the gDNA at this specific locus, nucleotide insertion or deletion were generated by the NHEJ pathway leading to either a less functional tRNA and/or a decreased expression of the tRNA. Another reason for targeting the acceptor arm was that, in human genome, there are two encoding Sec-tRNA^[Ser]Sec^ genes, *TRNAU1* and *TRNAU2* (in chromosome 19 and 22, respectively) which mostly differs in this region, as illustrated in Figure 1C. Even though *TRNAU2* is a pseudogene that does not express any Sec-tRNA^[Ser]Sec^ in cells [21], we thought that is was wise to design a CRISPR specific of *TRNAU1* gene. In addition, we calculated the MIT guide specificity score of our sgRNA targeting the *TRNAU1* gene (Position: chr19:45478589-45478611). The obtained MIT value of 99 in a 0–100 scale indicates a particularly low expected off-target effect.

Another specificity of our experimental design consisted in delivering Cas9-sgRNA RNP using CRISPR-Cas9-Viral-Like-Particles, referred to as Nanoblades [32]. In this system, different components from various viruses are dismantled in several pieces and are co-expressed in 293T producer cells by co-transfection of the respective plasmids. The stoichiometry between the five plasmids has been optimized [32] to produce large quantities of efficient CRISPR-VLPs. In these VLPs, the Cas9 is fused to the MLV Gag gene via a proteolytic linker. Therefore, the Cas9-sgRNA is released from the viral Gag protein within the particle and therefore delivered to the transduced cells. The pseudotyping with envelope proteins VSV-G and BaEVRless provides a wide tropism for the entry of the VLPs. Here, two different CRISPR-VLPs were produced with distinct sgRNAs, one targeting the *TRNAU1* gene and the other the *EMX-1* endogenous cellular gene as an internal control [32]; this was referred to as tRNA and EMX VLPs, respectively.

### 3.2. CRISPR-Cas9-VLPs Induced Mutations in TRNAU1 Gene with High Efficiency in Various Cell Lines

**Figure 3:**
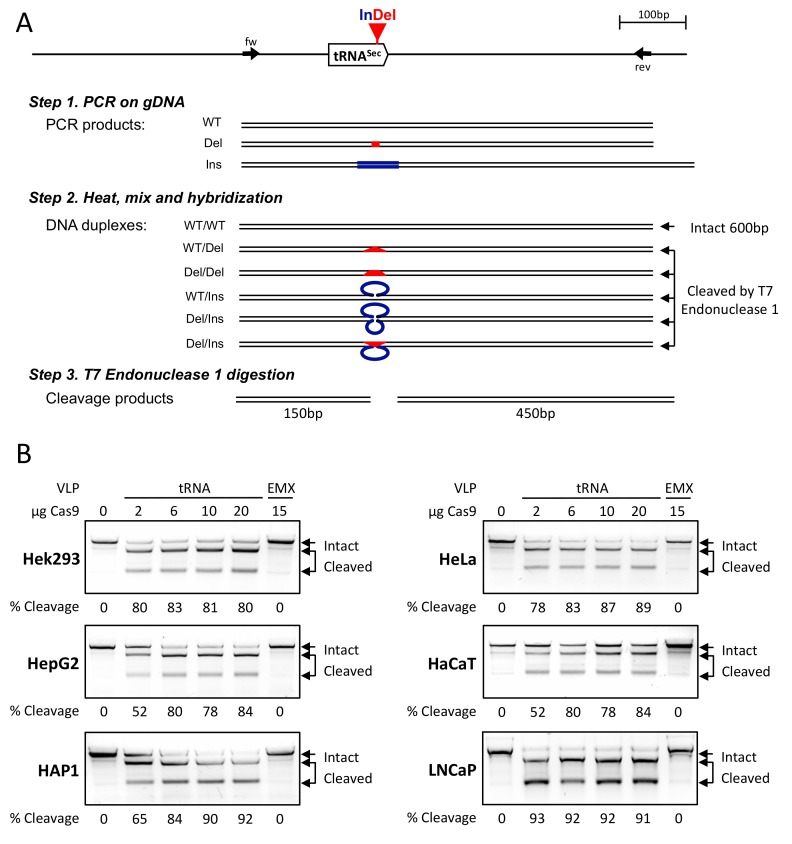
Evaluation of tRNA^[Ser]Sec^ gene editing activities by CRISPR-Cas9-VLPs used in various cell lines with T7 endonuclease 1 mismatch assay. (**A**) Illustration of this assay applied to detect Cas9 induced mutations. The gDNA was extracted from cells treated with Cas9-sgRNA and used for a polymerase chain reaction (PCR) amplification of the tRNA^[Ser]Sec^ gene using forward (fw) and reverse (rev) primers. The PCR product (fragment of approximately 600 bp) is composed of a mixture between the original wild-type (WT) and the Cas9 induced mutated sequences. The deletions (Del) and insertions (Ins) are represented in red and blue, respectively. The PCR products were heated, mixed and re-hybridized before addition of the T7 endonuclease 1, which cuts when heteroduplexes are formed during annealing (i.e., WT/Del, Del/Del, WT/Ins, Del/Ins and Del/Ins). The size of the digestion products were 150 and 450 bp. (**B**) The six different cell lines were treated with increasing amount CRISPR-Cas9-VLPs, targeting either tRNA^[Ser]Sec^ or EMX gene. Four days post-treatment, the cells were harvested, and their gDNA was analyzed with T7 endonuclease 1 assay. An aliquot of the reaction was run on an 2.5% agarose gel. The cleavage efficiency of the PCR fragment was calculated as the percentage of cleaved products relative to all fragments in each lane. The positions of the intact and cleaved DNAs are indicated by arrows.

**Figure 4:**
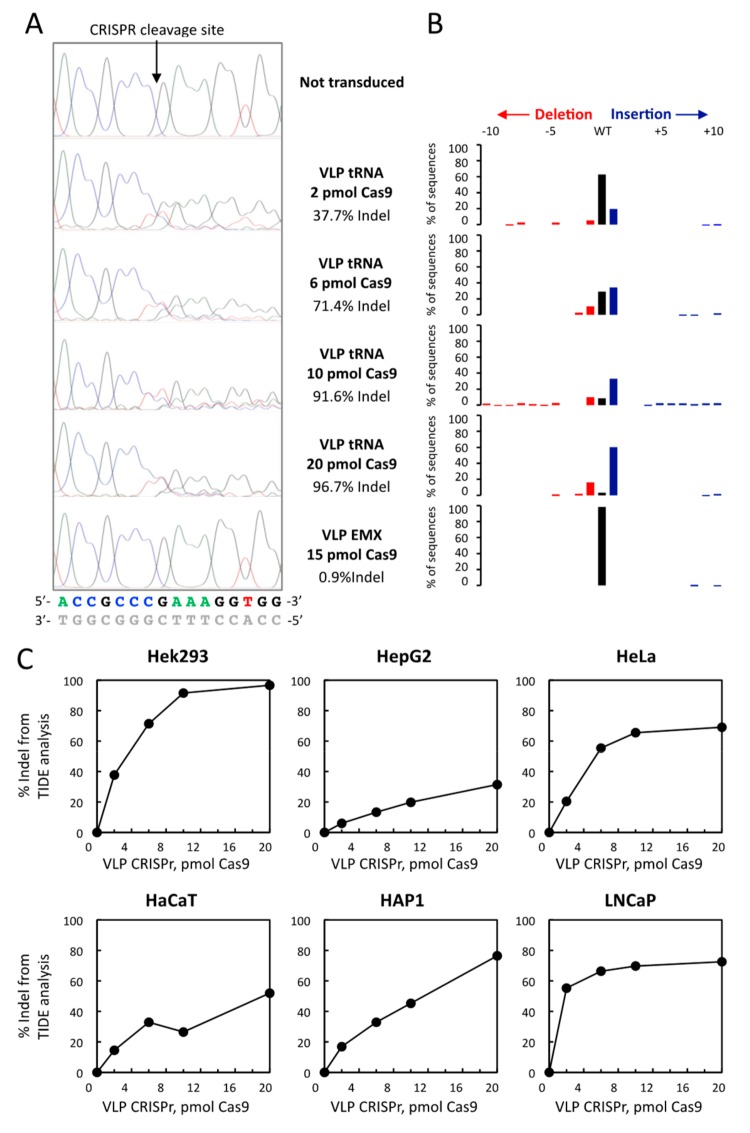
Evaluation of tRNA^[Ser]Sec^ gene editing efficiency by CRISPR-Cas9-VLPs with TIDE analysis. (**A**) Sanger sequencing of gDNA from Hek293 cells treated or not with tRNA or EMX VLPs. The PCR fragments encompassing the tRNA^[Ser]Sec^ gene used in Figure 2 for T7 endonuclease 1 mismatch assay were sequenced. The traces (.ab1 files) and the associated WT sequence are shown with A (green), T (red), G (black), C (blue). The complementary sequence is shown in light grey. The Indel score (%) was calculated from the resulting percentage of WT sequence found with TIDE software analysis. (**B**) Histogram representation of the TIDE results. The profiles of all insertion and deletion (Indel) in the treated samples are shown with deletions in red, insertions in blue, and WT sequences in black. (**C**) The genome editing efficiency was calculated with TIDE software for the six different cell lines treated with increasing amount CRISPR-Cas9-VLPs.

In the present study, we selected five cell lines that are commonly used in selenium biology, Hek293, HepG2, HaCaT, HeLa, and LNCaP, which originate from kidney, liver, skin, ovary and prostate, respectively. The other one (HAP1) that is derived from a patient with chronic myeloid leukemia is haploid and therefore widely used in CRISPR experiments. The Cas9 concentration was measured in the produced tRNA and EMX VLPs (Figure S1). The six cell lines were transduced identically using increasing amounts of tRNA or the control EMX VLPs. Four days after transduction, the cells were harvested and their gDNA was purified and analyzed either by T7 endonuclease 1 mismatch detection assay (Figure 3 and Reference [32]) or by Sanger sequencing followed by TIDE analysis (Figure 4 and Reference [34]).

As illustrated in Figure 3, the T7 endonuclease 1 mismatch detection assay is commonly used to evidence Cas9 induced mutations in gDNA. Although mostly qualitative, it allows the comparison of different experimental parameters such as the quantity of VLPs used for transduction. When the NHEJ repair system modifies the gDNA by inserting or deleting nucleotides at the Cas9 cutting locus, it generates a cutting site for the T7 endonuclease 1. Therefore, the efficiency of cleavage on the re-hybridized PCR product is directly correlated to the number of Cas9-induced mutations that have occurred. As observed in Figure 3B, at the highest concentration of tRNA VLPs, the cleavage efficiency of the 600 bp DNA into a 150 and a 450 bp fragments was higher than 80% in all of the six cell lines analyzed. As a control experiment, the transduction of EMX VLPs did not induce mutations at the *TRNAU1* locus of the gDNA in any of the cell lines studied. In order to determine the amount of VLPs required to reach the maximum Indel scores in different cell lines, we performed a dose–response analysis. Importantly, even with the highest amount of VLPs delivered, we did not observe any adverse effects on the growth and viability of the cell lines studied, as revealed by cell counting. In four cases, including HepG2, HAP1, HeLa, and HaCaT, we noticed an increase in cleavage efficiency in response to the amount of tRNA VLPs used for transduction. In the other two, namely Hek293 and LNCaP, the cleavage efficiency reached a plateau at the lowest concentration of tRNA VLPs added. However, due to the characteristics of the T7 endonuclease 1 assay, we could not compare the genome editing efficiencies between these different cell lines.

We then performed the analysis of the gDNA from the *TRNAU1* gene by TIDE software, which provides more quantitative results [34]. Based on the comparison of two Sanger sequences, the software performs a sequence trace decomposition from which the efficiency of genome editing can be estimated. Thus, the Indel score is inferred by the resulting percentage of differences from the wild-type (WT) sequence. A typical analysis is shown in Figure 4A,B for Hek293 cells in which different amounts of VLPs were transduced. When compared to the non-transduced cell line, the percentage of the Indel score increased with the quantity of Cas9-sgRNA in a dose-dependent manner to reach a maximal value of almost 100% in Hek293 cells. This result was highly specific since the EMX VLPs gave a background Indel of only 0,9% in a similar experiment. This analysis by TIDE software was applied to every cell lines and the Indel scores were plotted relative to the Cas9 concentration delivered (see Figure 4). This reflects that the optimal VLP amount might vary from one cell line to another. For the highest amount of Cas9 used (i.e., 20 pmol), the efficiencies of genome editing ranged as follows: Hek293 > HeLa, HAP1, LNCaP > HaCaT > HepG2.

Taken together, our results provide evidence that the sgRNA chosen here is particularly efficient to trigger mutations of the gDNA at the *TRNAU1* locus to reach an average of approximately 80% of genome editing. In addition, our data indicate that the VLPs have a broad tropism of transfection to deliver Cas9 in many cell lines.

### 3.3. Comparison of TIDE Analysis with the Sequencing of Indel in the Hek293 and HAP1 Cell Lines

**Figure 5:**
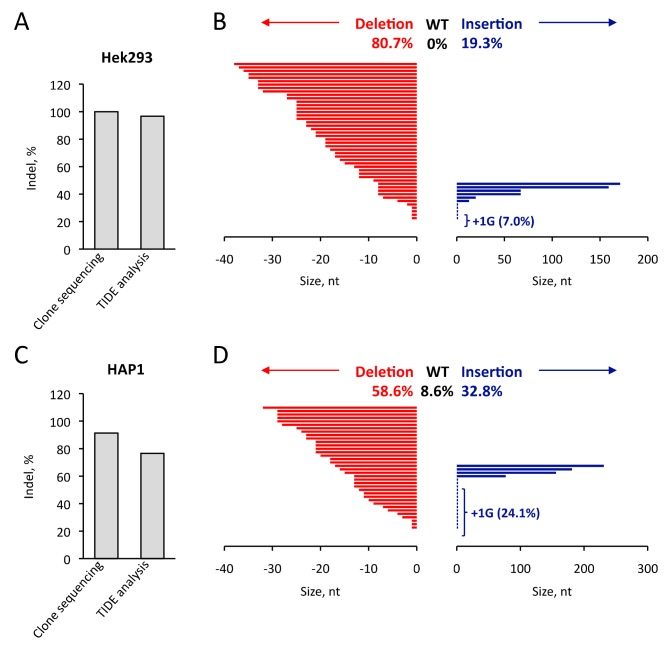
Analysis of the Cas9 induced mutations found in Hek293 and HAP1 cell lines. Genomic DNAs extracted from Hek293 and HAP1 cells treated with 20 pmol of Cas9 tRNA VLPs were used as a template for PCR amplification and cloning into a PCRII cloning vector. The sequences of the plasmid DNAs obtained from 57 and 58 colonies (Hek293 et HAP1, respectively) were used to calculate total genome editing efficiency (deletion plus insertion) at the TRNAU1 locus. This efficiency was compared to the one obtained by TIDE in Figure 4, for Hek293 (**A**) and HAP1 (**C**) cells. After alignment with the WT sequence, the size of the deleted (red) or inserted (blue) nucleotides are indicated in a histogram for Hek293 (**B**) and HAP1 (**D**) cells.

When comparing results from Figure 3 and Figure 4, it appears that the analyses by TIDE software provide more quantitative results than those obtained with the T7 endonuclease 1 mismatch detection assay. However, it has been reported that TIDE analysis slightly underestimates the actual genome editing as revealed by targeted next-generation sequencing (NGS). To receive further insight into the efficiency of our CRISPR design, we cloned the PCR products encompassing the *TRNAU1* gene in a TA cloning vector and sequenced the plasmids purified from isolated colonies. The cloning was performed after delivering the highest dose of tRNA VLPs in Hek293 and HAP1 cells and a total of 57 and 58 inserts were sequenced. In Hek293 cells, we inferred an Indel score of 100% since none of the WT sequence was retrieved in the sequenced clones and this was in agreement with the Indel score of 96.7% given by TIDE analysis (see Figure 5A). A similar set of results was also obtained with HAP1 (see Figure 5C,D), with the noticeable difference that several WT sequences were recovered (8.4%). Therefore, the Indel score deducted from sequencing (91,4%) was moderately higher than the one obtained from TIDE analysis (76.5%). Taken together, our data indicate that the genuine efficiency of genome editing is slightly underestimated by TIDE analysis and they validate accurately the correct design of our CRISPR strategy (Figure 5A,C).

In both cases, when looking at individual clones, the repartition of Indel was strongly in favor of deletions rather than insertions (see Figure 5B,D and S2). None of our clones had a single point mutation at the cleavage site. Interestingly, a significant number of our clones (7.0% in Hek2393, 24.1% in HAP1) have the same +1G insertion (Figure S2). This unexpected over representation may result from either a preferred NHEJ repair or a cellular selection for mutated tRNAs that could still be functional.

### 3.4. Selenium Levels and tRNA VLP Treatment Altered the Levels of tRNA^[Ser]Sec^

**Figure 6:**
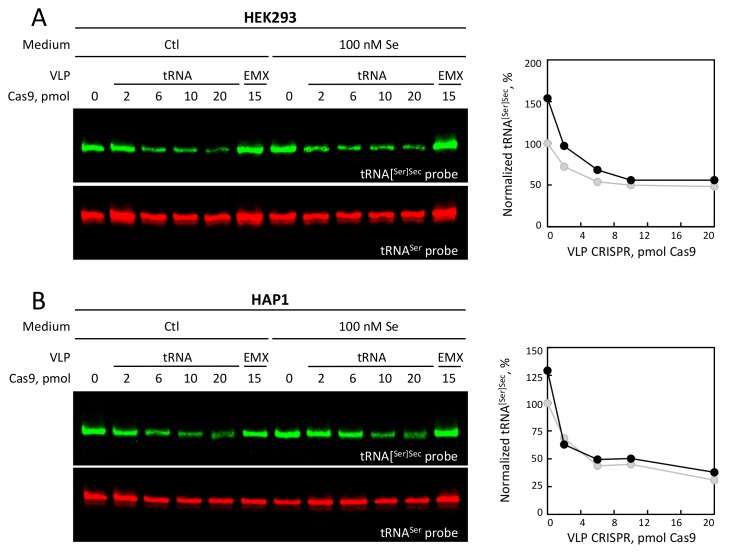
Evolution of tRNA^[Ser]Sec^ expression levels in response to the CRISPR-Cas9-VLP treatments and/or addition of selenium (100 nM) in Hek293 (**A**) and HAP1 (**B**) cell lines. Cells were treated with increasing amounts of CRISPR-Cas9-VLPs, targeting either tRNA^[Ser]Sec^ or EMX gene. Four days after transduction, cells were grown for another four days with or without 100 nM Se in the culture medium. Total RNAs were extracted and 15 μg were analyzed by northern blot using tRNA^[Ser]Sec^ (green) and tRNA^Ser^ (red) DNA probes for hybridization. The levels of tRNA^[Ser]Sec^ were normalized over the ones of tRNA^Ser^ and expressed relative to the first lane, which was set to 100%. In the right panels, the decrease of tRNA^[Ser]Sec^ levels was plotted as a function of Cas9 amount in control conditions (Ctl, grey circles) or with a 100 nM Se concentration in the medium (black circles).

The aim of this study was to modulate selenoprotein expression by either down-regulating the expression levels of tRNA^[Ser]Sec^ or to affect its function by modifying the 3′-side of its acceptor arm. To investigate this issue, we performed northern blot analysis with total RNAs extracted from Hek293 and HAP1 cells treated with tRNA or EMX VLPs. Infrared fluorescent oligonucleotides were used to probe the tRNA^[Ser]Sec^ (IR800) and the tRNA^Ser^ (IR700) represented as green and red in Figure 5, respectively. The expression level of tRNA^[Ser]Sec^ was normalized to the level of tRNA^Ser^. It should be noted that in these experiments, the concentration of selenium was adjusted to 100 nM and compared with unsupplemented medium. With both Hek293 and HAP1 cell lines, we observed an increase of tRNA^[Ser]Sec^ abundance in response to selenium supplementation. This induction of steady state levels of tRNA^[Ser]Sec^ by selenium supplementation was previously observed in human myeloid leukemia (HL-60) cells and rat mammary tumor (RMT) with 127 (10 ng/mL) and 109 nM (8.5 ng/mL) of selenium added, respectively [28]. However, the transduction of the tRNA VLPs triggered a decrease in tRNA^[Ser]Sec^ levels of 50 and 70% in Hek293 and HAP1 cells, respectively (see Figure 6).

In Hek293 cells, the efficiency of genome editing was close to 100%, indicating that the remaining 50% of the tRNA^[Ser]Sec^ detected by northern blot should bear mutations. These mutations in the *TRNAU1* gene do not seem to inhibit the transcription nor the maturation of tRNA^[Ser]Sec^ but they could affect the stability of the tRNA. Indeed, it has been reported that the removal of 3′-trailer and the addition of CCA end are important steps in the quality control stage of tRNAs [37,38]. In any case, our data demonstrated that our Cas9-sgRNA design leads to a strong reduction of tRNA^[Ser]Sec^ levels in Hek293 cells.

Interestingly, a more pronounced decrease in tRNA^[Ser]Sec^ levels was observed in HAP1 than in Hek293 cells (Figure 6), even though genome editing efficiency was slightly lower in HAP1 (Indel score of 91,4%). This data suggest that the effects of genome editing could vary amongst different cell lines and this can be explained by several features. First, HAP1 cell lines are haploids while Hek293 cells have between four to five copies of chromosome 19, the chromosome which bears the *TRNAU1* gene [39] as many immortalized or cancerous cell lines used in research laboratories contain numerous chromosomal copies. Secondly, the endogenous concentration of the tRNA^[Ser]Sec^ and the requirement for selenoprotein activities may greatly vary between cell lines. As a consequence, the balance between the modifications of the tRNA^[Ser]Sec^ gene and the maintenance of cell viability should be specific to every cell line.

### 3.5. Selenoprotein Levels Are Differently Affected by CRISPR-Cas9-VLPs Amongst Cell Lines

**Figure 7:**
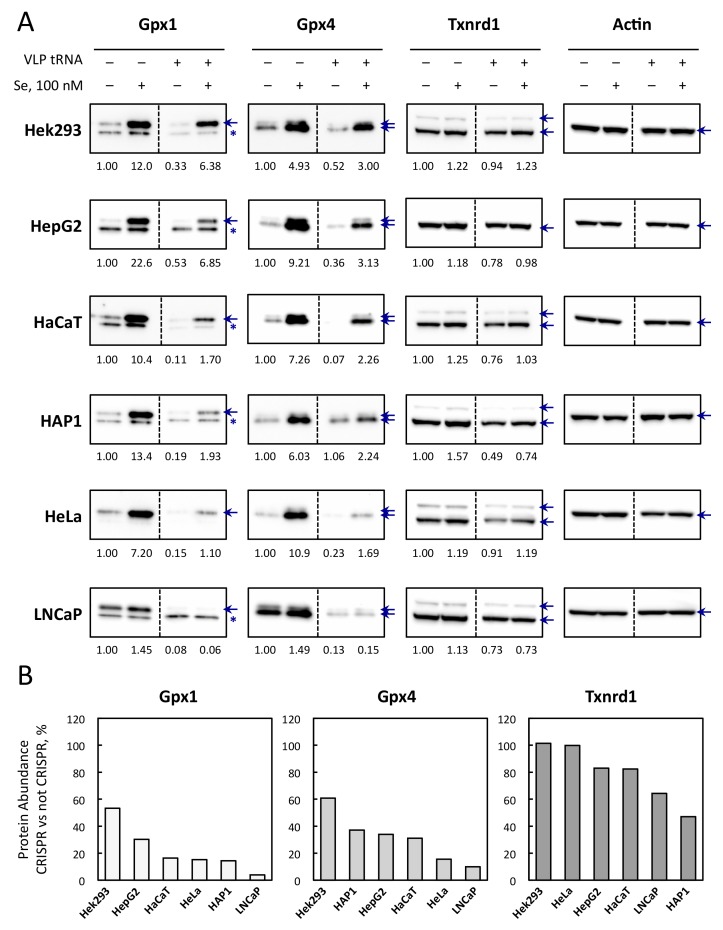
Evolution of Gpx1, Gpx4, and Txnrd1 protein levels in response to the VLP treatment and/or addition of selenium (100 nM) in various cell lines. (**A**) The six different cell lines were treated, or not, with 20 pmol of Cas9 tRNA VLPs and amplified for another four days with or without 100 nM Se in the culture medium. Protein extracts from the harvested cells were analyzed for selenoprotein levels by performing western blots with specific antibodies against Gpx1, Gpx4, Txnrd1 and Actin. The selenoproteins are indicated by blue arrows. According to the manufacturer, Gpx1 gives an non-specific band (indicated by an asterisk) below the correct one. Gpx4 has two isoforms, mitochondrial and cytoplasmic, respectively. Depending on cell lines, Txnrd1 has several isoforms. The quantifications were normalized to the intensity of the signal for actin and expressed relative to the non-VLP treated condition in control medium, set as 1. (**B**) The relative decrease of Gpx1, Gpx4, and Txnrd1 protein abundance in response to 20 pmol of Cas9 tRNA VLPs treatment and in presence of 100 nM Se was plotted for the six different cell lines.

Next, we looked at the expression of ubiquitous selenoproteins, Gpx1, Gpx4, and Txnrd1, which ranked differently in the selenoprotein hierarchy in response to selenium fluctuation. As reported in [36] and shown in Figure 7, the sensitivity to selenium supplementation ranged as follows: Gpx1 > Gpx4 > Txnrd1 in most cells. In several cell lines, such as HeLa, Gpx4 was slightly more sensitive to selenium variation than Gpx1. Thus, we investigated whether the down-regulation of tRNA^[Ser]Sec^ could affect the expression of these well-characterized selenoproteins in response to selenium supplementation and in different cellular models (Figure 7A). Interestingly, we observed that in every cell line tested here, the down-regulation of tRNA^[Ser]Sec^ had a similar effect on selenoprotein expression, with Gpx1 being more sensitive than Gpx4, and Txnrd1 being the least responsive (Figure 7B). Importantly, even though Txnrd1 was poorly regulated by selenium supplementation, its expression was significantly affected by the VLP treatment in the majority of the cell lines tested.

The results obtained with Hek293 cells were particularly informative, since the genome editing reached almost 100% at the *TRNAU1* locus. The observation that these cells are still able to insert a selenocysteine and produce selenoproteins at significant levels indicate that the tRNA^[Ser]Sec^ was still functional, at least partially, with mutations in the acceptor stem. Changes in selenium levels induce a prioritized synthesis of selenoprotein, and this phenomenon is linked to changes in tRNA^[Ser]Sec^ levels [28]. Therefore, it seems rational that reducing tRNA^[Ser]Sec^ levels and/or its activity would induce a similar hierarchy than the decrease of selenium levels. Taken together, our data provide evidence that selenoprotein expression can be significantly decreased by targeting the *TRNAU1* gene in different cell lines.

### 3.6. Selenoprotein mRNA Levels Are Not Affected by tRNA^[Ser]Sec^ Down-Regulation in Hek293 and HAP1

**Figure 8:**
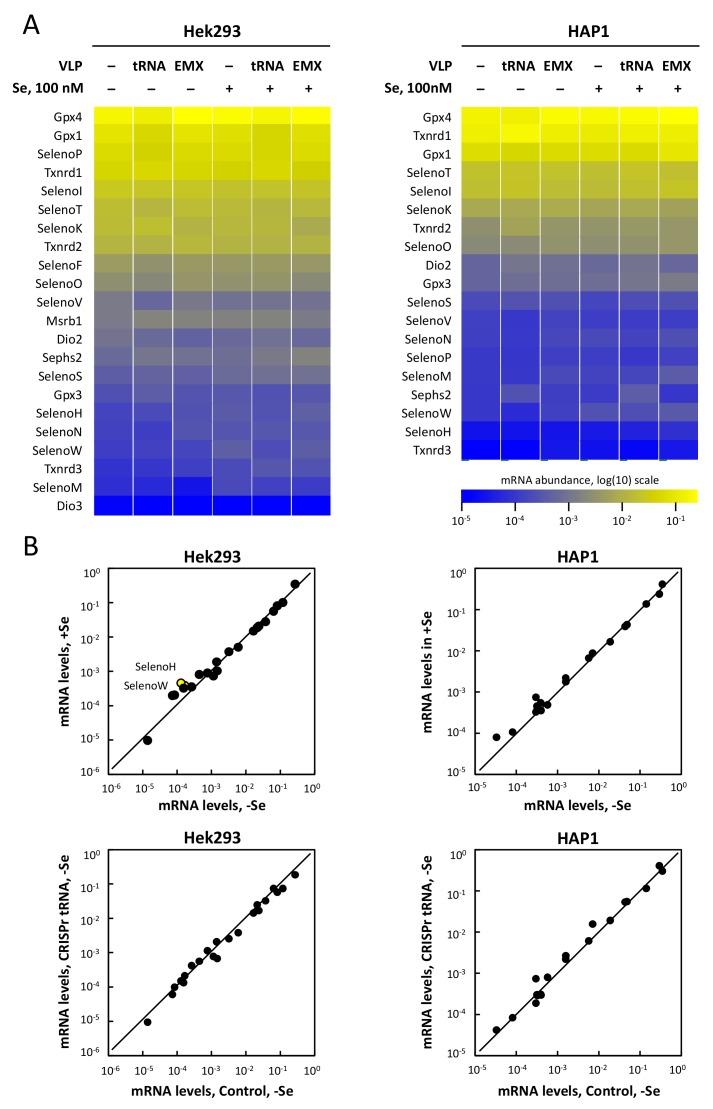
Evolution of selenoprotein mRNA levels in response to the CRISPR-Cas9-VLP treatments and/or addition of selenium (100 nM) in Hek293 and HAP1 cell lines. (**A**) The RNA extracts used in Figure 6 were also used for RT-qPCR analysis to measure selenoprotein and housekeeping mRNA levels. The geometrical mean of five housekeeping genes (Hpcb, Rps13, rRNA 18S, Hrpt, and Gapdh) was used to normalize mRNA abundance. Selenoprotein mRNA levels are represented in logarithmic scale in a heatmap. The values are given in Table S2 and Table S3. (**B**) To validate the stability of steady state levels of selenoprotein mRNAs, the values obtained in control conditions (no VLP, no added Se) were plotted as a function of values obtained with 100 nM Se (top histograms) or with tRNA VLP treated conditions (bottom histograms) in a logarithmic scale. For clarity reasons, only mRNAs for which a minimum of two-fold difference (and p-value below 0.05) were represented in yellow circle and labeled in the graphs.

When the UGA-recoding efficiency is altered, the UGA codon can then be seen as a premature stop codon by the nonsense mediated decay (NMD). For example, the knockdown of SECISBP2 was shown to reduce the levels of several selenoprotein mRNAs, including SelenoH, SelenoT, Txnrd2, and Gpx1 [40]. Additionally, in cells from patients with mutant forms of SECISBP2, several transcripts were significantly reduced (SelenoH, SelenoT, and SelenoW) while others were preserved or even upregulated (SelenoO, SelenoI, SelenoM, and SelenoK) [41]. Since we observed a decrease in selenoprotein levels, it was therefore important to verify whether the reduced levels of tRNA^[Ser]Sec^ could have an impact on the landscape of selenoprotein transcripts. As illustrated in Figure 8, total RNAs extracted from Hek293 and HAP1 treated, or not, with tRNA or EMX VLPs, and supplemented, or not, with 100 nM selenium, were evaluated for the expression levels of the 25 selenoprotein mRNAs. These data were normalized to the levels of five housekeeping transcripts (HspcB, rPS13,18S rRNA, Hprt, Gapdh) used as a reference. First, we confirmed that in Hek293 and HAP1 cell lines, selenium supplementation did not dramatically alter the level of expression of selenoprotein transcripts. Only SelenoH and SelenoW were significantly different between supplemented and unsupplemented extracts in Hek293 cells (Figure 8B, left panel). These data are in agreement with our recently published work [36], where we reported only marginal variations of selenoprotein mRNAs in response to selenium supplementation in various cell lines, including Hek293, HepG2, HaCaT, and LNCaP. In the present work, we further evaluated whether the selenoprotein transcripts were affected by the tRNA VLP treatments. We found no significant variation of these transcripts in response to VLP treatments (EMX or tRNA) indicating that selenoprotein transcripts were not targeted by the NMD following the decrease in levels of tRNA^[Ser]Sec^ and this situation occurred independently from the addition of selenium in the medium.

### 3.7. The Overexpression of tRNA^[Ser]Sec^ Allowed the Recovery of Selenoprotein Expression in VLP Treated Cells

**Figure 9:**
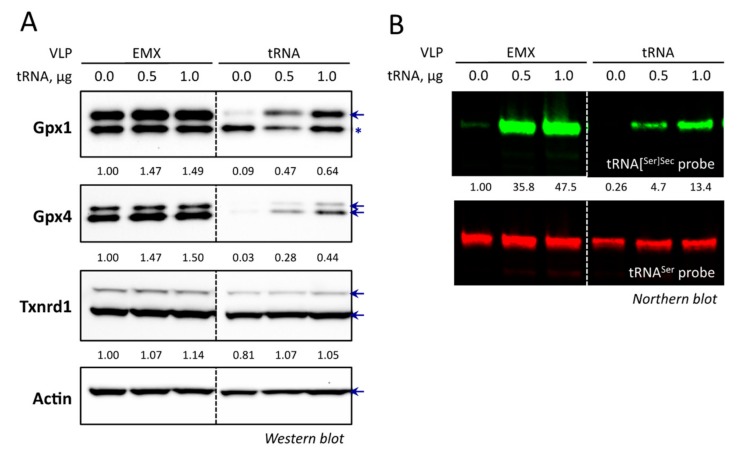
The selenoprotein levels can be restored in Hek293 cells treated with tRNA VLPs by the overexpression of WT tRNA^[Ser]Sec^. Hek293 cells treated with EMX or tRNA VLPs were transfected with two concentrations of tRNA^[Ser]Sec^ encoding plasmid in a 6-well plate. The cells were harvested and analyzed for selenoprotein expression levels by western blots (**A**) or for tRNA^[Ser]Sec^ levels by northern blot (**B**) similarly to Figure 6 and Figure 7, respectively.

In order to validate the design of our experimental strategy and rule out a potential off-target effect, it was important to confirm that the decrease of tRNA^[Ser]Sec^ is actually directly responsible for the down-regulation of selenoprotein levels. Therefore, we performed rescue experiments where the wild-type *TRNAU1* gene was transfected in VLP-treated cells. It has been previously shown that tRNA^[Ser]Sec^ overexpression had a moderate stimulatory effect on selenoprotein levels in wild-type cells [42,43]. As illustrated in Figure 9, when we increased the levels of tRNA^[Ser]Sec^ in Hek293 cells treated with the control VLPs, this led to higher expression of Gpx1, Gpx4, and Txnrd1. In cells treated with the tRNA VLPs, even though we had a lower transfection efficiency (data not shown), the overexpression of the tRNA led to an efficient recovery of selenoprotein expression to a level similar to the wild type (Figure 9A). By performing northern blotting experiments, we confirmed that the expression of the tRNA^[Ser]Sec^ was restored (Figure 9B). Taken together, these data confirmed that the effects observed with the VLPs are indeed due to Cas9 induced mutations (knock-out and mutations in the 3’ side of the acceptor arm) of the *TRNAU1* gene. In the future, it will be important to determine the molecular mechanism put in place and, especially, how these mutant forms of the tRNA can still be able to be matured, modified, and charged with selenocysteine.

## 4. Discussion

### 4.1. Development of a Novel Method to Produce Cells Lines With Reduced Selenoprotein Levels

Changing the selenium concentration of the culture medium is the most common method to modulate the expression of the selenoproteome. This results in significant differences in selenoprotein production with some being more expressed than others both in cultured cells and animals; this is referred to as the selenoprotein hierarchy [1,11,36,44,45]. However, changes in selenium levels can have other indirect cellular consequences, such as modulating potential trace element signaling pathways. Therefore, in order to precisely understand the function of selenoproteins in various mechanisms, there is certainly a need to develop alternative methods to modulate selenoprotein expression without changing the intracellular concentration of selenium. This was the global aim of this study, in which we describe a novel strategy to target the 3′-side of tRNA acceptor stem of the tRNA^[Ser]Sec^ by using a CRISPR/Cas9 approach based on delivery via a retroviral VLP. The Cas9 induced mutations in the gDNA locus induced functional disruptions that significantly altered tRNA expression and therefore reduced selenoprotein levels. The use of a VLP system allowed the efficient delivery of its contents to virtually any human cell line, as observed here and reported for other cell lines [32].

The tRNA^[Ser]Sec^ is one of the key components of the UGA recoding machinery by the ribosome. Present in only one copy in the human genome and required for the synthesis of all of the 25 selenoproteins, it is the ideal target to control selenoproteome expression. However, there is a fine balance between the modification of tRNA concentration or alteration of its function and the maintenance of cell viability. Indeed, the complete inactivation of the tRNA^[Ser]Sec^ gene is lethal in mice and even a conditional knockout in mice liver, endothelial cells, heart and skeletal muscle, and skin leads to premature animal death [24]. Next, in most of other animal models with tissue selective inactivation of the tRNA^[Ser]Sec^ gene, a strong phenotype was also observed, even though the mice remained viable. These data obtained in animals suggested that our first goal should be to reduce the tRNA^[Ser]Sec^ concentration to a level which is compatible with cell survival. This is why we have induced Cas9 mutations at the site near the 3′-end of the tRNA, as it seemed less detrimental for cell viability than in another region. On the other hand, it appeared that the level of genome editing efficiency did not completely match with the phenotype in all cell lines. However, the phenotype at the selenoprotein level seemed to be specific for every cell line tested and thus needed to be characterized every time in response to VLP-CRISPR transduction. In our hands, we were able to reduce between 50% and 90% of the production of Gpx1 and between 40% and 85% of that of Gpx4 in the six cell lines studied (Figure 7). Importantly, in every case, we observed that the expression of all selenoproteins retained their ability to be stimulated by selenium induction. A novelty of our experimental approach lies in the delivery mode of Cas9-sgRNA RNP by viral pseudoparticles instead of transfections or infection with CRISPR-coding viral vectors. In comparison with these commonly used methods, our novel strategy allows to fine tune the level of genome editing with minimal off-target cleavages of gDNA [32]. We validated the proof of concept of this method in six human cell lines by applying the same protocol of transduction. Indeed, the current version of the VLP-CRISPR has a wide tropism due to the presence of the two envelope proteins VSV-G and BaEVRless. Overall, we provide a versatile strategy to significantly reduce the levels of expression of selenoproteins in various human cells without altering their ability to respond to selenium concentrations in the medium.

Another conceivable strategy for down-regulating tRNA^[Ser]Sec^ levels is the use of small hairpin RNAs (shRNAs) gene silencing. To our knowledge, only one successful example of tRNA down-regulation by shRNAs has been reported in mammals and this was achieved by targeting the tRNA^Thr^ (CGU) that pairs to the ACG codon which is one of the rarest tRNAs in HeLa cells [46]. However, reduction of the tRNA^Thr^ (CGU) concentration remained of limited extent reaching only between a 25 to 40% decrease, depending on the nature of the shRNAs used. Moreover, the treatment with shRNAs targeting the anticodon loop of tRNA^Thr^ (CGU) was only effective for a limited period of time (48 h). In our case, the editing on the gDNA is a definite genetic event which allows the selection by cellular sub-cloning. Another major difference between a shRNA and CRISPR strategy to down-regulate tRNAs, lies in the fact that shRNA only reduces the concentration of the endogenous tRNA target whilst Cas9 induces multiple mutations in the gene locus that result in alterations of gene expression. As such, the targeting of the 3′-end of the tRNA^[Ser]Sec^ can induce changes at the level of RNA-PolIII transcription, 3′-trailer sequence removal, 3′-end CCA addition, post-transcriptional modifications and cloverleaf folding. Modifications of all of these steps will have effects on the ability of the tRNA to recode UGA into selenocysteine and its stability. Our data show that we obtained the right balance between modifications of the levels and function of the tRNA^[Ser]Sec^ gene and the maintenance of cell viability in our experimental design.

### 4.2. The Cas9-Induced Mutations in TRNAU1 Gene Lead to a Selective Down-Regulation of Selenoproteins to a Level Which Is Similar to Selenium Deficiency

As stated before, an increase in selenium concentration in the culture medium results in the activation of selenoproteins but to a level that varies greatly across them. Although well described, this so-called hierarchy is only partially understood at the molecular level. It is proposed that the tRNA^[Ser]Sec^, the SECIS and SECISBP2 are involved in this phenomenon [1,3]. Concerning the tRNA, its steady state level and modification at the wobble position U34 are sensitive to selenium changes. To our knowledge, this is the first report of tRNA modification by a CRISPR-Cas9 experimental strategy. Obviously, the number of copies of tRNA genes represents a major hurdle for this strategy as tRNAs are often coded by several genes. However, in our case, the tRNA^[Ser]Sec^ was present at only one functional copy in the human genome with the other copy having evolved towards a pseudogene. As observed by sequencing the Cas9-induced mutations of the gDNA at the TRNAU1 locus, the extent of genome editing ranged between 38-nt deletion and 171-nt insertion in Hek293 and between 32-nt deletion and 231-nt insertion in HAP1 cells. In Hek293 cells, genome editing attained nearly 100% efficiency, this means that every tRNA gene was expected to be hit by Cas9-sgRNA cleavage. However, the analysis by northern blot indicated that a significant level of full-length tRNA remained expressed in this Hek293 population. Therefore, this suggests that some mutations in the 3′-end of the tRNA can be tolerated without a significant loss of function. This was confirmed by the fact that these mutants were still responsive to changes in selenium concentration. Another interesting finding was that the selenoproteins are differently affected by the reduced levels and mutations of the tRNA^[Ser]Sec^. As such, the expression of Gpx1 was more sensitive than Gpx4 and Txnrd1 was the least responsive one. Thus, the hierarchy in selenoprotein expression is maintained after the transduction of tRNA VLPs and is similar in all points to what is observed upon changes in selenium concentration. These data validate nicely our experimental system as a novel alternative to study the regulation of the selenoproteome.

Since HAP1 cells are haploid, they represent a very convenient model for genome editing. In this cell line, all chromosomes are expected to be present in only one copy. Therefore, mutations and/or large deletions are expected to induce strong phenotypes on selenoprotein synthesis. In our hands, these deletions were viable in HAP1 suggesting that either selenoproteins are not essential in this cell line or that these mutants have retained a residual functional activity to maintain cell viability. Structure-function analyses of the TRNAU1 gene will now be possible with the many Cas9-induced mutants generated by our Cas9-sgRNA design. It should be noted that one mutation is overrepresented in HAP1 and it consists in a +1 insertion of a G at the Cas9 cutting site. This mutation is expected to change the 3′-side of the acceptor arm from CUUUCGGGCGCCA into CUUUC**G**GGGCCCA (underlined is the changed sequence, and in bold the inserted G). It will be of great interest to determine whether or not this mutation can affect any of the transcription, maturation, aminoacylation or stability of the tRNA^[Ser]Sec^. In any case, if a positive selection has occurred for this mutation over others in HAP1 cells, it certainly suggests that this modification is either silent or less detrimental than others. Alternatively, this could also be due to a repair bias in the insertion of a G rather than any another nucleotide by the NHEJ machinery. Since this machinery is different from one cell line to another, the Cas9-induced mutations are expected to be different from one model to another.

## 5. Conclusions

In this manuscript, we designed a novel method to reduce selenoprotein expression without changing the selenium concentration by targeting the human tRNA^[Ser]Sec^. This was rendered possible by the development of a CRISPR-Cas9-VLP that induced mutation at the 3′-end of the tRNA. This Cas9-sgRNA design was able to knock-down tRNA^[Ser]Sec^ expression to a level that was sufficient to significantly reduce selenoprotein levels while maintaining cell viability. Proof of concept of this method was validated in six human cell lines, namely Hek293, HepG2, HaCaT, HAP1, HeLa, and LNCaP. Overall, this method offers an original, novel alternative to study variations in the expression of the selenoproteome without affecting the intracellular level of selenium concentration.

## Supplementary Materials

The following are available online at https://www.mdpi.com/2073-4409/8/6/574/s1, Figure S1: Evaluation of Cas9 content in tRNA and EMX VLPs, Figure S2: Sequence alignment of the PCRII inserts shown in Figure 5B,D for Hek293 and HAP1 cell lines, respectively, Table S1: List of qPCR primers used in this study, Table S2: Values of Selenoprotein mRNA levels in response to the CRISPR-Cas9-VLP treatments and/or addition of selenium (100 nM) in HAP1 cell lines obtained by RT-qPCR, Table S3: Values of selenoprotein mRNA levels in response to the CRISPR-Cas9-VLP treatments and/or addition of selenium (100 nM) in Hek293 cell lines obtained by RT-qPCR.
