# Supplementary material for: A Versatile Strategy to Reduce UGA-Selenocysteine Recoding Efficiency of the Ribosome Using CRISPR-Cas9-Viral-Like-Particles Targeting Selenocysteine-tRNA[Ser]Sec Gene
Source: Cells. 2019 Jun 11;8(6):574. doi: 10.3390/cells8060574 (PMC6627462; doi:10.3390/cells8060574)

Figure S1: Quantification of the amount of Cas9 packaged into tRNA and EMX VLPs by dot blot analysis. Serial dilutions of the recombinant Cas9 were spotted onto a nitrocellulose membrane and used as a reference for the Cas9-containing VLPs.

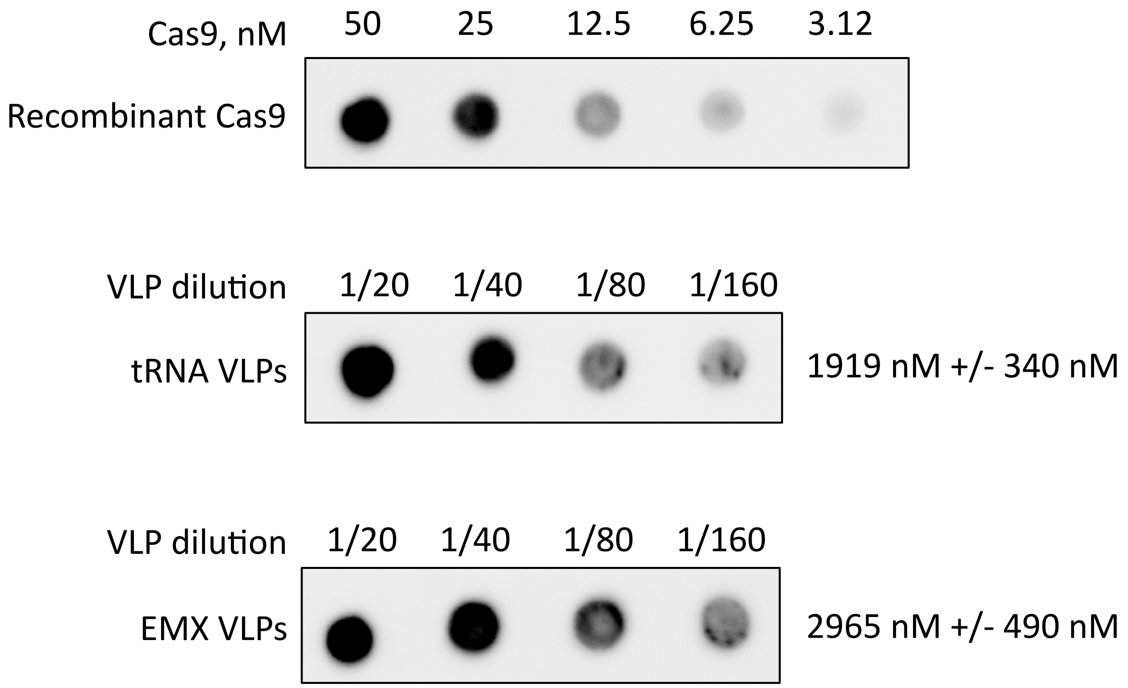

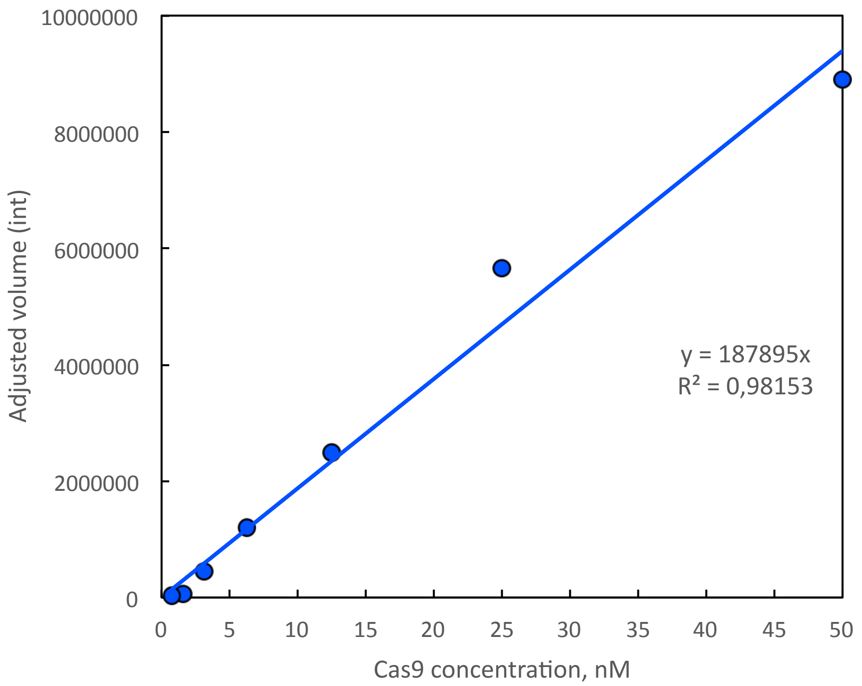

Supplement: Supplementary file 1 [file cells-08-00574-s001.zip › supplementary/Figure S1.pdf]
