# Supplementary material for: A Versatile Strategy to Reduce UGA-Selenocysteine Recoding Efficiency of the Ribosome Using CRISPR-Cas9-Viral-Like-Particles Targeting Selenocysteine-tRNA[Ser]Sec Gene
Source: Cells. 2019 Jun 11;8(6):574. doi: 10.3390/cells8060574 (PMC6627462; doi:10.3390/cells8060574)
Supplement: Supplementary file 1 [file cells-08-00574-s001.zip › supplementary/Figure S2.pdf]

Figure S2: Sequence alignment of the PCRII inserts shown in Figure 5B and D for Hek293 and HAP1 cell lines, respectively. The WT sequence is used as a reference with the region targeted by the sgRNA shown in grey and the pam sequence underlined. sequences A1-A46 : deletions in Hek293 (red); sequences A47-A57 : Insertions in Hek293 (blue); sequences B1-B5 : WT in HAP1; sequences B6-B39 : deletions in HAP1 (red); sequences A40-A58 : Insertions in HAP1 (blue).

HEK293 Deletions

```
>WT     ATAAGTAAGATTTAAGGCGCTTAGTTACTACCGCCCGAAAGGTGGAATTGAACCACTCTGTCGCTAGACAGCTACAGGTTTGAA
>A1     ATAAGTAAGATTCAAGGCGCTTAGTTACTACCGCC-GAAAGGTGGAATTGAACCACTCTGTCGCTAGACAGCTACAGGTTTGAA
>A2     ATAAGTAAGATTCAAGGCGCTTAGTTACTACCGCC-GAAAGGTGGAATTGAACCACTCTGTCGCTAGACAGCTACAGGTTTGAA
>A3     ATAAGTAAGATTCAAGGCGCTTAGTTACTACCGCC-TTGTGGTGGAATTGAACCACTCTGTCGCTAGACAGCTACAGGTTTGAA
>A4     ATAAGTAAGATTCAAGGCGCTTAGTTACTACCGCCC--AAGGTGGAATTGAACCACTCTGTCGCTAGACAGCTACAGGTTTGAA
>A5     ATAAGTAAGATTCAAGGCGCTTAGTTACTACCG----AAAGGTGGAATTGAACCACTCTGTCGCTAGACAGCTACAGGTTTGAA
>A6     ATAAGTAAGATTCAAGGCGCTTAGTTAC-------CGAAAGGTGGAATTGAACCACTCTGTCGCTAGACAGCTACAGGTTTGAA
>A7     ATAAGTAAGATTCAAGGCGCTTAGTTACTACCGCC--------TAAATTGAACCACTCTGTCGCTAGACAGCTACAGGTTTGAA
>A8     ATAAGTAAGATTCAAGGCGCTTAGTTACTACCGCC--------TAAATTGAACCACTCTGTCGCTAGACAGCTACAGGTTTGAA
>A9     ATAAGTAAGATTCAAGGCGCTTAGTTACTAC--------AGGTGGAATTGAACCACTCTGTCGCTAGACAGCTACAGGTTTGAA
>A10    ATAAGTAAGATTCAAGGCGCTTAGTTACT--------AAAGGTGGAATTGAACCACTCTGTCGCTAGACAGCTACAGGTTTGAA
>A11    ATAAGTAAGATTCAAGGCGCTTAGTTA---------GAAAGGTGGAATTGAACCACTCTGTCGCTAGACAGCTACAGGTTTGAA
>A12    ATAAGTAAGATTCAAGGCGCTTAGTTACTACCGCC------------TTGTACCACTCTGTCGCTAGACAGCTACAGGTTTGAA
>A13    ATAAGTAAGATTCAAGGCGCTTAGTTA------------AGGTGGAATTGAACCACTCTGTCGCTAGACAGCTACAGGTTTGAA
>A14    ATAAGTAAGATTCAAGGCGCTTAGTTA------------AGGTGGAATTGAACCACTCTGTCGCTAGACAGCTACAGGTTTGAA
>A15    ATAAGTAAGATTCAAGGCGCTT-------------CGAAAGGTGGAATTGAACCACTCTGTCGCTAGACAGCTACAGGTTTGAA
>A16    ATAAGTAAGATTCAAGGCGCTTA---------------AAGGTGGAATTGAACCACTCTGTCGCTAGACAGCTACAGGTTTGAA
>A17    ATAAGTAAGATTCAAGGCGCT----------------AAAGGTGGAATTGAACCACTCTGTCGCTAGACAGCTACAGGTTTGAA
>A18    ATAAGTAAGATTCAAGGCGC-----------------AAAGGTGGAATTGAACCACTCTGTCGCTAGACAGCTACAGGTTTGAA
>A19    ATAAGTAAGATTCAAGGCGC-----------------AAAGGTGGAATTGAACCACTCTGTCGCTAGACAGCTACAGGTTTGAA
>A20    ATAAGTAAGATTCAAGGCGCTTAGT------------------GGAATTGAACCACTCTGTCGCTAGACAGCTACAGGTTTGAA
>A21    ATAAGTAAGATTCAAGGCGCTTAGTTACT-------------------TGAACCACCCTGTCGCTAGACAGCTACAGGTTTGAA
>A22    ATAAGTAAGATTCAAGGCGCTTAGTTA-------------------ATTGAACCACTCTGTCGCTAGACAGCTACAGGTTTGAA
>A23    ATAAGTAAGATTCAAGGCGCTTAG---------------------AATTGAACCACTCTGTCGCTAGACAGCTACAGGTTTGAA
>A24    ATAAGTAAGATTCAAGGCGCTTAGTTACTAC---------------------GCACTCTGTCGCTAGACAGCTACAGGTTTGAA
>A25    ATAAGTAAGATTCAAGGCGCTTAGT-----------------------TGAACCACTCTGTCGCTAGACAGCTACAGGTTTGAA
>A26    ATAAGTAAGATTCAAGGCGCTTAGT-----------------------TGAACCACTCTGTCGCTAGACAGCTACAGGTTTGAA
>A27    ATAAGTAAGATTCAAGGCGCTTAGTTACT-------------------TGAACCACTCTGTCGCTAGACAGCTACAGGTTTGAA
>A28    ATAAGTAAGATTCAAGGCGCTTAGTT----------------------TGAACCACTCTGTCGCTAGACAGCTACAGGTTTGAA
>A29    ATAAGTAAGATTCAAGGCGCTTAGTTACTA-------------------------CTCTGTCGCTAGACAGCTACAGGTTTGAA
>A30    ATAAGTAAGATTCAAGG-------------------------TGGAATTGAACCACTCTGTCGCTAGACAGCTACAGGTTTGAA
>A31    ATAAGTAAGATTCAAGGCGCT-------------------------TTTGAACCACTCTGTCGCTAGACAGCTACAGGTTTGAA
>A32    ATAAGTAAGATTCAAGGCG-------------------------GAATTGAACCACTCTGTCGCTAGACAGCTACAGGTTTGAA
>A33    ATAAGTAAGATTCAAGGCG-------------------------GAATTGAACCACTCTGTCGCTAGACAGCTACAGGTTTGAA
>A34    ATAAGTAAGATTCAAGGCGCTTAGTTAC-------------------------CACTCTGTCGCTAGACAGCTACAGGTTTGAA
>A35    ATAAGTAAGATTCAAGGCGCTTAG-------------------------GAACCACTCTGTCGCTAGACAGCTACAGGTTTGAA
```

```
>A36  ATAAGTAAGATTCAAGGCGC---------------------------TTGAACCACTCTGTCGCTAGACAGCTACAGGTTTGAA
>A37  ATAAGTAAGATTCAAGG---------------------------GAATTGAACCACTCTGTCGCTAGACAGCTACAGGTTTGAA
>A38  ATAAGAAAGG--------------------------------TGGAATTGAACCACTCTGTCGCTAGACAGCTACAGGTTTGAA
>A39  ATAAGTAAGATT---------------------------------AATTGAACCACTCTGTCGCTAGACAGCTACAGGTTTGAA
>A40  ATAAGTAAGATTCAAG---------------------------------GAACCACTCTGTCGCTAGACAGCTACAGGTTTGAA
>A41  ATAAGTAAGATTCAAGGCGCTTA-----------------------------------TGTCGCTAGACAGCTACAGGTTTGAA
>A42  ATAAGTAAGATTCAAGGCGCTTA-----------------------------------TGTCGCTAGACAGCTACAGGTTTGAA
>A43  ATAAGTAAGATTCAA------------------------------------ACCACTCTGTCGCTAGACAGCTACAGGTTTGAA
>A44  ATAAGTAAGATTC-------------------------------------AACCACTCTGTCGCTAGACAGCTACAGGTTTGAA
>A45  ATAAGTAAGATTC---------------------------------ATTGAACCACTCTGTCGCTAGACAGCTACAGGTTTGAA
>A46  ATAAGTAAGA--------------------------------------TGAACCACTCTGTCGCTAGACAGCTACAGGTTTGAA
```

HEK293 Insertions

>WT ATAAGTAAGATTTAAGGCGCTTAGTTACTACCGCCCGAAAGGTGGAATTGAACCACTCTGTCGCTAGACAGCTACAGGTTTGAA
>A47 ATAAGTAAGATTCAAGGCGCTTAGTTACTACCGCCCCGAAAGGTGGAATTGAACCACTCTGTCGCTAGACAGCTACAGGTTTGAA
>A48 ATAAGTAAGATTCAAGGCGCTTAGTTACTACCGCCCCGAAAGGTGGAATTGAACCACTCTGTCGCTAGACAGCTACAGGTTTGAA
>A49 ATAAGTAAGATTCAAGGCGCTTAGTTACTACCGCCCCGAAAGGTGGAATTGAACCACTCTGTCGCTAGACAGCTACAGGTTTGAA
>A50 ATAAGTAAGATTCAAGGCGCTTAGTTACTACCGCCTTGAAAGGTGGAATTGAACCACTCTGTCGCTAGACAGCTACAGGTTTGAA
>A51 ATAAGTAAGATTCAAGGCGCTTAGTTACCACCGCCCCGAAAGGTGGAATTGAACCACTCTGTCGCTAGACAGCTACAGGTTTGAA
>A52 ATAAGTAAGATTCAAGGCGCTTAGTTACTACCGCTTAGTTACGACAGAGTGGAATTGAACCACTCTGTCGCTAGACAGCTACAGGTTTGAA
>A53 ATAAGTAAGATTCAAGGCGCTTAGTTACTACCGCCTTCAATTCAGAAAGGTGGAATCGAACCACTCTGTCGCTAGACAGCTACAGGTTTGAA
>A54 ATAAGTAAGATTCAAGGCGCTTAGTTACTACCGCCCCTTGTCAGTTCTCTTGGATGAGCAACTGTGTTTGGTCATGGGGGCAAGGCACTGACCTTGGAGCTCCGAAAGGTGGAAT
TGAACCACTCTGTCGCTAGACAGCTACAGGTTTGAA
>A55 ATAAGTAAGATTCAAGGCGCTTAGTTACTACCGCCCCTTGTCAGTTCTCTTGGATGAGCAACTGTGTTTGGTCATGGGGGCAAGGCACTGACCTTGGAGCTCCGAAAGGTGGAAT
TGAACCACTCTGTCGCTAGACAGCTACAGGTTTGAA
>A56 ATAAGTAAGATTCAAGGCGCTTAGTTACTACCACAGAGCTGAACCTTCCTTTAGACAGAGCAGATTTGAAACAGCCTATTTGTGCAGTTTCCAGTTGGAGATTTCAATCGCTTTG
AGACCAAATGTAGAAAAGGAAACATCTTCGTATAAAAACTAGACAGAATCATTCTCAGAAACTACTTTGTGATGTGTGCGTTCAACTCAAGGTGGAATTGAACCACTCTGTCGCTAGACAG
CTACAGGTTTGAA
>A57 ATAAGTAAGATTCAAGGCGCTTAGTTACTACCGTGGGGCTGGGCGCCCGTGATCCCAGCTACTCAGGAGGCTGAGGCAGGAGAATCACATGAACCCAGAGGGCAGAGGTTGCAGT
GAGCTGAGATCTCACCATTGAACTCCAGCCTGGGAGACAGAGCGAGACTCTGTCTCAAAACAAACGAAAGGTGGAATTGAACCACTCTGTCGCTAGACAGCTACAGGTTTGAA

HAP1 WT

```
>WT   ATAAGTAAGATTTAAGGCGCTTAGTTACTACCGCCCGAAAGGTGGAATTGAACCACTCTGTCGCTAGACAGCTACAGGTTTGAA
>B1   ATAAGTAAGATTTAAGGCGCTTAGTTACTACCGCCCGAAAGGTGGAATTGAACCACTCTGTCGCTAGACAGCTACAGGTTTGAA
>B2   ATAAGTAAGATTTAAGGCGCTTAGTTACTACCGCCCGAAAGGTGGAATTGAACCACTCTGTCGCTAGACAGCTACAGGTTTGAA
>B3   ATAAGTAAGATTTAAGGCGCTTAGTTACTACCGCCCGAAAGGTGGAATTGAACCACTCTGTCGCTAGACAGCTACAGGTTTGAA
>B4   ATAAGTAAGATTTAAGGCGCTTAGTTACTACCGCCCGAAAGGTGGAATTGAACCACTCTGTCGCTAGACAGCTACAGGTTTGAA
>B5   ATAAGTAAGATTTAAGGCGCTTAGTTACTACCGCCCGAAAGGTGGAATTGAACCACTCTGTCGCTAGACAGCTACAGGTTTGAA
```

HAP1 Deletions

```
>WT   ATAAGTAAGATTTAAGGCGCTTAGTTACTACCGCCCGAAAGGTGGAATTGAACCACTCTGTCGCTAGACAGCTACAGGTTTGAA
>B6   ATAAGTAAGATTTAAGGCGCTTAGTTACTACCGC-CGAAAGGTGGAATTGAACCACTCTGTCGCTAGACAGCTACAGGTTTGAA
>B7   ATAAGTAAGATTTAAGGCGCTTAGTTACTACC-ACCGAAAGGTGGAATTGAACCACTCTGTCGCTAGACAGCTACAGGTTTGAA
>B8   ATAAGTAAGATTTAAGGCGCTTAGTTACTACC-ACCGAAAGGTGGAATTGAACCACTCTGTCGCTAGACAGCTACAGGTTTGAA
>B9   ATAAGTAAGATTTAAGGCGCTTAGTTACTACCGCC---AAGGTGGAATTGAACCACTCTGTCGCTAGACAGCTACAGGTTTGAA
>B10  ATAAGTAAGATTTAAGGCGCTTAGTTACTAC----CGAAAGGTGGAATTGAACCACTCTGTCGCTAGACAGCTACAGGTTTGAA
>B11  ATAAGTAAGATTTAAGGCGCTTAGTTACTACCGCCC------TGGAATTGAACCACTCTGTCGCTAGACAGCTACAGGTTTGAA
>B12  ATAAGTAAGATTTAAGGCGCTTAGTTACTACCGCCCG-------GAATTGAACCACTCTGTCGCTAGACAGCTACAGGTTTGAA
>B13  ATAAGTAAGATTTAAGGCGCTTAGTTACTA---------AGGTGGAATTGAACCACTCTGTCGCTAGACAGCTACAGGTTTGAA
>B14  ATAAGTAAGATTTAAGGCGCTTAGTTACTACCGC----------GAATTGAACCACTCTGTCGCTAGACAGCTACAGGTTTGAA
>B15  ATAAGTAAGATTTAAGGCGCTTAGTTACTACCG-----------GAATTGAACCACTCTGTCGCTAGACAGCTACAGGTTTGAA
>B16  ATAAGTAAGATTTAAGGCGCTTAG-----------CGAAAGGTGGAATTGAACCACTCTGTCGCTAGACAGCTACAGGTTTGAA
>B17  ATAAGTAAGATTTAAGGCGCTTA------------CGAAAGGTGGAATTGAACCACTCTGTCGCTAGACAGCTACAGGTTTGAA
>B18  ATAAGTAAGATTTAAGGCGCTTAG-------------AAAGGTGGAATTGAACCACTCTGTCGCTAGACAGCTACAGGTTTGAA
>B19  ATAAGTAAGATTTAAGGCGCTTAG-------------AAAGGTGGAATTGAACCACTCTGTCGCTAGACAGCTACAGGTTTGAA
>B20  ATAAGTAAGATTTAAGGCGCTTAGTTACTACCGCC-------------TTAACCACTCTGTCGCTAGACAGCTACAGGTTTGAA
>B21  ATAAGTAAGATTTAAGGCGCTTAGTTACTA---------------TTTTGAACCACTCTGTCGCTAGACAGCTACAGGTTTGAA
>B22  ATAAGTAAGATTTAAGGCGCTTAGTTACTACCGCCC----------------CCACTCTGTCGCTAGACAGCTACAGGTTTGAA
>B23  ATAAGTAAGATTTAAGGCG-----------------GCAAGGTGGAATTGAACCACTCTGTCGCTAGACAGCTACAGGTTTGAA
>B24  ATAAGTAAGATTTAAGGCGCTTAGT------------------GGAATTGAACCACTCTGTCGCTAGACAGCTACAGGTTTGAA
>B25  ATAAGTAAGATTTAAGGCGCTTAGT------------------GGAATTGAACCACTCTGTCGCTAGACAGCTACAGGTTTGAA
>B26  ATAAGTAAGATTTAAGGCGCTTAGT--------------------AATTGAACCACTCTGTCGCTAGACAGCTACAGGTTTGAA
>B27  ATAAGTAAGATTTAAGGCGCTT---------------------GGAATTGAACCACTCTGTCGCTAGACAGCTACAGGTTTGAA
>B28  ATAAGTAAGATTTAAGGCGCTTAGTT---------------------TTGAACCACTCTGTCGCTAGACAGCTACAGGTTTGAA
>B29  ATAAGTAAGATTTAAGGCGCTTAGTTACTACCGCC---------------------TCTGTCGCTAGACAGCTACAGGTTTGAA
>B30  ATAAGTAAGATTTAAGGCGCTTAGTTACTACCGCC---------------------TCTGTCGCTAGACAGCTACAGGTTTGAA
>B31  ATAAGTAAGATTTAAGGCGCTTA-----------------------ATTGAACCACTCTGTCGCTAGACAGCTACAGGTTTGAA
>B32  ATAAGTAAGATTTAAGGCGCTTA-----------------------ATTGAACCACTCTGTCGCTAGACAGCTACAGGTTTGAA
>B33  ATAAGTAAGATTTAAGGCGCTTA------------------------TTGAACCACTCTGTCGCTAGACAGCTACAGGTTTGAA
>B34  ATAAGTAAGATTTAAGGCG-------------------------GAATTGAACCACTCTGTCGCCAGACAGCTACAGGTTTGAA
>B35  ATAAGTAAGA----------------------------AAGGTGGAATTGAACCACTCTGTCGCTAGACAGCTACAGGTTTGAA
>B36  ATAAGTAAGATTTAAGGCGCTTA-----------------------------CCACTCTGTCGCTAGACAGCTACAGGTTTGAA
>B37  ATAAGTAAGATTTAAGGCGCTTA-----------------------------CCACTCTGTCGCTAGACAGCTACAGGTTTGAA
>B38  ATAAGTAAGATTTAAGGCGCTTA-----------------------------CCACTCTGTCGCTAGACAGCTACAGGTTTGAA
>B39  ATAAGTAAGATTTAAGGCGCTTA--------------------------------CTCTGTCGCTAGACAGCTACAGGTTTGAA
```

HAP1 Insertions

>WT ATAAGTAAGATTTAAGGCGCTTAGTTACTACCGCCCGAAAGGTGGAATTGAACCACTCTGTCGCTAGACAGCTACAGGTTTGAA

>B40 ATAAGTAAGATTCAAGGCGCTTAGTTACTACCGCCCCGAAAGGTGGAATTGAACCACTCTGTCGCTAGACAGCTACAGGTTTGAA

>B41 ATAAGTAAGATTCAAGGCGCTTAGTTACTACCGCCCCGAAAGGTGGAATTGAACCACTCTGTCGCTAGACAGCTACAGGTTTGAA

>B42 ATAAGTAAGATTCAAGGCGCTTAGTTACTACCGCCCCGAAAGGTGGAATTGAACCACTCTGTCGCTAGACAGCTACAGGTTTGAA

>B43 ATAAGTAAGATTCAAGGCGCTTAGTTACTACCGCCCCGAAAGGTGGAATTGAACCACTCTGTCGCTAGACAGCTACAGGTTTGAA

>B44 ATAAGTAAGATTCAAGGCGCTTAGTTACTACCGCCCCGAAAGGTGGAATTGAACCACTCTGTCGCTAGACAGCTACAGGTTTGAA

>B45 ATAAGTAAGATTCAAGGCGCTTAGTTACTACCGCCCCGAAAGGTGGAATTGAACCACTCTGTCGCTAGACAGCTACAGGTTTGAA

>B46 ATAAGTAAGATTCAAGGCGCTTAGTTACTACCGCCCCGAAAGGTGGAATTGAACCACTCTGTCGCTAGACAGCTACAGGTTTGAA

>B47 ATAAGTAAGATTCAAGGCGCTTAGTTACTACCGCCCCGAAAGGTGGAATTGAACCACTCTGTCGCTAGACAGCTACAGGTTTGAA

>B48 ATAAGTAAGATTCAAGGCGCTTAGTTACTACCGCCCCGAAAGGTGGAATTGAACCACTCTGTCGCTAGACAGCTACAGGTTTGAA

>B49 ATAAGTAAGATTCAAGGCGCTTAGTTACTACCGCCCCGAAAGGTGGAATTGAACCACTCTGTCGCTAGACAGCTACAGGTTTGAA

>B50 ATAAGTAAGATTCAAGGCGCTTAGTTACTACCGCCCCGAAAGGTGGAATTGAACCACTCTGTCGCTAGACAGCTACAGGTTTGAA

>B51 ATAAGTAAGATTTAAGGCGCTTAGTTACTACCGCCCCGAAAGGTGGAATTGAACCACTCTGTCGCTAGACAGCTACAGGTTTGAA

>B52 ATAAGTAAGCTTTAAGGCGCTTAGTTACTACCGCCCCGAAAGGTGGAATTGAACCACTCTGTCGCTAGACAGCTACAGGTTTGAA

>B53 ATAAGTAAGATTTAAGGCGCTTAGTTACTACCGCCCCGAAAGGTGGAATTGAACCACTCTGTCGCTAGACAGCTACAGGTTTGAA

>B54 ATAAGTAAGATTTAAGGCGCTTAGTTACTACCGCCCAGAAAGGTGGAATTGAACCACTCTGTCGCTAGACAGCTACAGGTTTGAA

>B55 ATAAGTAAGATTTAAGGCGCTTAGTTACTACCGCCCTGCAAGCAGATATTTGACCTCTTTGAGGCCTTCGTTGGAAACGGGATTTCTTCATAGAACGCTAGAAAGAAGAATACGAAAGGTGGGATTGAACCACTCTGTCGCTAGACAGCTACAGGTTTGAA

>B56 ATAAGTAAGATTTAAGGCGCTTAGTTACTACCGCCCGGCCATTCAGAGCCCAGGGCTAACAGGTGTCTGGACCTTTCTTTCTAGGCCCACAGAAGCTCTCTGGGCACCCGGGGCCTCTTGAGGCCAGGTGGGCTTTCTGGACATGCAGGCGTCCTGGGGCCCCCAGCATCTGTGTCCACATTTTGTACCCGCGAAAGGTGGAATTGAACCACTCTGTCGCTGGACAGCTACAGGTTTGAA

>B57 ATAAGTAAGATTTAAGGCGCTTAGTTACTACCGCCCCCCAATTTTCTTTTGTAGCCAAGTCTATACTTGTACTTGGTGCTCTAACAGCTTACTGTTTGTGTGAAAATTTTAGCGATCTCCCTGTGCCTCATTTCCATCTTTCTCTCAAACTATCTCTGCCTCTTTTTTCTACTTTCTTCTCATCTGTCCTTACATGTTATTTCATGTTTATTCAGTCGAAAGGTGGAATTGAACCACTCTGTCGCTAGACAGCTACAGGTTTGAA

>B58 ATAAGTAAGATTTAAGGCGCTTAGTTACTACCGCCCAATTCATTCTTTAGCAATTTGTTCATTAATTTTCTTTTCTTCAAGTAATTTTGCTTGATTTGCAAAGAAAGTTTCAGCTTCTTGTCTTATTTGGCTAATTTGTTTACCTAGTTCTTTTTTTTCTTCATTTGAAACAGATTTTAGTTGATTTTGTAATTTTGCTAAATAGCTATCTTTACCAAAAAAAGCATTTTTGGCTAATTTTAAATCTTCTAAATTGTTAATGACAAAGAAAGGTGGAATTGAACCACTCTGTCGCTAGACAGCTACAGGTTTGAA
