# Supplementary material for: A Versatile Strategy to Reduce UGA-Selenocysteine Recoding Efficiency of the Ribosome Using CRISPR-Cas9-Viral-Like-Particles Targeting Selenocysteine-tRNA[Ser]Sec Gene
Source: Cells. 2019 Jun 11;8(6):574. doi: 10.3390/cells8060574 (PMC6627462; doi:10.3390/cells8060574)
Supplement: Supplementary file 1 [file cells-08-00574-s001.zip › supplementary/Table S1.pdf]

Table S1. List of qPCR primers used in this study

Target Sequence	GenBank Accession Number		Sequence	Product length	Efficiency
**Dio1**	NM_001039716.1	fwd	CACTGCCTGAGAGGCTCTACATA	75	1,728
		rev	TGTAGTTCCAAGGGCCAGAT		
**Dio2**	NM_000793.5	fwd	CCTCCTCGATGCCTACAAAC	82	1,713
		rev	TCCTTCTGTACTGGAGACATGC		
**Dio3**	NM_001362	fwd	AACTCCGAGGTGGTTCTGC	60	2,000
		rev	TTGCGCGTAGTCGAGGAT		
**Gpx1**	NM_000581	fwd	GCAACCAGTTTGGGCATCAG	123	1,989
		rev	CGTTCACCTCGCACTTCTCG		
**Gpx2**	NM_002083.2	fwd	GTCCTTGGCTTCCCTTGC	67	ND
		rev	TGTTCAGGATCTCCTCATTCTG		
**Gpx3**	NM_002084.3	fwd	GGGGACAAGAGAAGTCGAAGA	116	2,000
		rev	GCCAGCATACTGCTTGAAGG		
**Gpx4**	NM_001039848	fwd	TGGGAAATGCCATCAAGTGG	108	1,935
		rev	GGTCCTTCTCTATCACCAGGGG		
**Gpx6**	NM_182701.1	fwd	AATGGAGAAAAAGAACAGAAGGTC	75	ND
		rev	TGAGCCCAAAAGATCAGAGG		
**MSRB1**	NM_016332.2	fwd	GAGGTTTTCCAGAATCACTTTGA	104	2,000
		rev	GGCCATGGAGACGAGTGT		
**SELENOH**	NM_170746.2	fwd	GCTTCCAGTAAAGGTGAACCCG	190	2,000
		rev	ACCCAAATCTCCCTACGACAGG		
**SELENOI**	NM_033505.1	fwd	GGTCGTCATGGCTGGCTA	88	2,000
		rev	TGGATTGGTATCCACAGCAC		
**SELNOK**	NM_021237.3	fwd	ATCTGATTCCAGATATGATGATGG	72	1,627
		rev	TGATTGATTCTACCCATTCTTCG		
**SELNOM**	NM_080430	fwd	TCCCGATGAGCCTCCTGTTG	201	1,924
		rev	ATGGAATGTCCTGCGTGACG		
**SELENON**	NM_206926	fwd	AAGGGCAAGGAGGTCATCATCC	195	2,000
		rev	AGGGGAGAACCAAAAGGGGAAG		
**SELENOO**	NM_031454	fwd	GAGGAGTTTGACGCCGAGTTC	165	1,893
		rev	GCTCAGCAAGTAGAAGGTGTTTGTG		
**SELENOP**	NM 005410.2	fwd	GGAGCTGCCAGAGTAAAGCA	72	1,853
		rev	ACATTGCTGGGGTTGTCAC		
**SELENOS**	NM_203472.1	fwd	AAACGGAAATCGGACAGAAA	65	2,000
		rev	CCTCCTTCACCAGACAACG		
**SELENOT**	NM_016275.3	fwd	TCCAGATTTGTGTTTCCTGAGG	75	2,000
		rev	CTGGGTACCGCTGGCTAATA		
**SELENOV**	NM_182704.1	fwd	AAAAGGTAGCCGGCAAGG	62	1,832
		rev	TTCAGCCCTTCTCATCATCC		
**SELENOW**	NM_003009	fwd	GCCGTCCGAGTCGTTTATTGTG	149	1,996
		rev	GCTACCATCACTTCAAAGAACCCG		
**SELENOF**	NM_004261.3	fwd	GTTGTTGGCGACTGTGCTT	64	2,000
		rev	GCATGCCTCCGATGAAAA		
**SEPHS2**	NM_012248	fwd	CCACGGACTTCTTTTACCCCTTG	156	2,000
		rev	GTTCCTCCTCACTCATACTCTGGC		
**TXNRD1**	NM_003330	fwd	CCTTATCATCATTGGAGGTGGCTC	106	1,878
		rev	AAGAGGGGTGGGAGTGACAAAGTC		
**TXNRD2**	NM_006440	fwd	CACATCTACGCCATTGGTGACG	142	1,927
		rev	AGACGGTCGTGGGAACATTGTC		
**TXNRD3**	XM_001129642.1	fwd	AAGGAAATTTGGCTGGGAAT	74	1,967
		rev	GGTTCTGAATCGCTTTTGTCA		
**HPRT**	NM_000194	fwd	TGACACTGGCAAAACAATGCA	94	1,826
		rev	GGTCCTTTTCACCAGCAAGCT		
**HSPCB**	NM_007355	fwd	TCTGGGTATCGGAAAGCAAGC	80	1,969
		rev	GTGCACTTCCTCAGGCATCTTG		
**RPS13**	NM_001017	fwd	CGAAAGCATCTTGAGAGGAACA	87	1,907
		rev	TCGAGCCAAACGGTGAATC		
**18S rRNA**	NT_167214.1	fwd	AGAAACGGCTACCACATCCA	169	1,937
		rev	CACCAGACTTGCCCTCCA		
**GAPDH**	NM_002046	fwd	CGACAGTCAGCCGCATCTT	63	1,838
		rev	CCCCATGGTGTCTGAGCG		
