# Supplementary material for: A Versatile Strategy to Reduce UGA-Selenocysteine Recoding Efficiency of the Ribosome Using CRISPR-Cas9-Viral-Like-Particles Targeting Selenocysteine-tRNA[Ser]Sec Gene
Source: Cells. 2019 Jun 11;8(6):574. doi: 10.3390/cells8060574 (PMC6627462; doi:10.3390/cells8060574)
Supplement: Supplementary file 1 [file cells-08-00574-s001.zip › supplementary/Table S2.pdf]

Table S2. Values of Selenoprotein mRNA levels in response to the CRISPR-Cas9-VLP treatments and/or addition of selenium (100 nM) in HAP1 cell lines obtained by RT-qPCR. The geometrical mean of five housekeeping genes (Hpcb, Rps13, rRNA 18S, Hrpt and Gapdh) was calculated and used to normalize mRNA abundance.

**VLP**	**No**		**tRNA**		**EMX**		**No**		**tRNA**		**EMX**		**No VLP/tRNA VLP**		**Ctl/100 nMSe (No VLP)**	
**Selenium**	**Ctl**		**Ctl**		**Ctl**		**100 nM**		**100 nM**		**100 nM**					
	Ave	SD	Ave	SD	Ave	SD	Ave	SD	Ave	SD	Ave	SD	Fold change	p value	Fold change	p value
Selenoprotein genes																
**Gpx4**	3,46E-01	5,70E-02	3,02E-01	5,48E-02	4,68E-01	2,13E-02	4,11E-01	1,89E-02	4,65E-01	6,53E-02	5,49E-01	6,49E-02	1,147	0,011	0,842	0,1251
**TXNRD1**	2,92E-01	7,73E-03	4,07E-01	1,13E-01	2,67E-01	6,04E-03	2,38E-01	9,19E-04	2,93E-01	6,20E-02	2,76E-01	2,80E-02	0,718	0,204	1,229	0,0356
**Gpx1**	1,41E-01	3,02E-03	1,15E-01	1,19E-02	1,31E-01	6,12E-03	1,36E-01	1,12E-03	1,26E-01	8,09E-03	1,97E-01	1,25E-02	1,228	0,075	1,035	0,0873
**SELENOT**	4,80E-02	1,07E-02	5,48E-02	2,53E-03	5,09E-02	7,62E-03	4,28E-02	5,62E-04	5,40E-02	1,25E-03	4,55E-02	1,60E-04	0,876	0,300	1,121	0,3163
**SELENOI**	4,34E-02	8,85E-08	5,35E-02	3,37E-04	4,10E-02	7,53E-04	3,88E-02	9,49E-04	4,79E-02	6,80E-03	5,42E-02	1,86E-03	0,813	0,008	1,119	0,0458
**SELENOK**	1,89E-02	2,42E-04	1,91E-02	4,14E-03	2,10E-02	3,41E-03	1,65E-02	2,30E-03	1,90E-02	6,19E-03	1,47E-02	1,36E-03	0,987	0,472	1,144	0,2067
**TXNRD2**	7,09E-03	1,52E-03	1,56E-02	1,31E-02	9,07E-03	4,04E-04	8,77E-03	2,46E-03	1,01E-02	1,53E-03	9,45E-03	2,06E-03	0,455	0,244	0,809	0,1200
**SELENOO**	5,69E-03	2,42E-05	6,11E-03	9,82E-05	8,19E-03	2,64E-04	6,64E-03	2,76E-04	7,74E-03	1,41E-03	9,49E-03	7,84E-04	0,931	0,064	0,857	0,0702
**DIO2**	1,59E-03	1,43E-04	2,67E-03	5,65E-04	2,25E-03	1,54E-03	1,76E-03	5,54E-05	2,56E-03	3,59E-04	1,62E-03	7,48E-05	0,595	0,138	0,899	0,2128
**GPX3**	1,58E-03	5,22E-04	2,19E-03	9,29E-04	2,19E-03	2,92E-04	2,19E-03	7,54E-04	2,69E-03	1,03E-03	3,50E-03	2,74E-05	0,720	0,140	0,720	0,0833
**SELENOS**	5,72E-04	1,40E-04	7,99E-04	1,69E-04	7,18E-04	1,30E-04	4,87E-04	1,39E-05	6,47E-04	2,43E-05	7,16E-04	1,03E-04	0,715	0,244	1,173	0,2903
**SELENOV**	3,99E-04	3,93E-04	2,96E-04	1,63E-04	4,46E-04	1,08E-04	3,55E-04	5,09E-05	3,50E-04	4,60E-05	3,62E-04	5,11E-05	1,348	0,320	1,124	0,4428
**SELENON**	3,90E-04	1,60E-05	3,09E-04	1,04E-04	5,23E-04	6,49E-05	5,48E-04	7,78E-05	4,56E-04	2,65E-04	6,78E-04	1,68E-04	1,262	0,257	0,713	0,1270
**SELENOP**	3,23E-04	3,11E-05	2,88E-04	2,00E-04	3,81E-04	1,17E-05	3,80E-04	2,39E-05	2,99E-04	2,26E-05	4,23E-04	6,79E-05	1,123	0,408	0,851	0,0287
**SELENOM**	3,17E-04	8,41E-05	3,01E-04	7,75E-05	5,55E-04	9,63E-05	4,53E-04	9,88E-05	4,99E-04	9,09E-05	1,06E-03	6,30E-04	1,055	0,087	0,700	0,0243
**SEPHS2**	3,00E-04	6,97E-05	7,38E-04	4,89E-05	3,68E-04	4,04E-05	3,27E-04	2,14E-05	1,07E-03	4,43E-04	2,86E-04	1,87E-05	0,406	0,060	0,915	0,2833
**SELENOW**	2,99E-04	3,85E-04	1,88E-04	1,35E-04	3,99E-04	4,02E-08	7,46E-04	1,09E-04	6,98E-04	1,01E-04	9,82E-04	3,47E-04	1,589	0,322	0,401	0,1312
**SELENOH**	8,11E-05	3,06E-07	8,35E-05	1,00E-05	9,21E-05	3,96E-09	1,06E-04	8,89E-06	1,39E-04	8,52E-06	2,27E-04	1,01E-04	0,971	0,392	0,761	0,0747
**TXNRD3**	3,32E-05	9,72E-06	4,19E-05	1,16E-11	9,49E-05	2,72E-05	7,95E-05	4,77E-05	5,27E-05	2,55E-05	1,00E-04	5,16E-05	0,793	0,213	0,417	0,1672
Reference genes																
**18S**	2,15E+01	1,90E+00	2,18E+01	2,25E-01	2,64E+01	1,20E+00	2,52E+01	9,38E-04	2,42E+01	1,41E+00	2,21E+01	6,55E-05	0,987	0,427	0,855	0,1123
**GAPDH**	1,48E+00	8,36E-02	1,31E+00	3,00E-01	1,04E+00	1,01E-01	1,22E+00	1,15E-01	1,18E+00	2,57E-01	1,09E+00	7,71E-03	1,131	0,321	1,219	0,1546
**HSPCB**	6,86E-01	1,09E-01	7,33E-01	5,17E-02	7,24E-01	1,03E-01	6,07E-01	7,82E-01	7,91E-01	2,70E-01	7,44E-01	8,14E-02	0,935	0,226	1,129	0,4479
**HPRT**	2,64E-01	2,49E-02	3,06E-01	2,19E-02	2,44E-01	3,99E-03	2,82E-01	3,91E-03	2,35E-01	8,14E-03	2,18E-01	2,25E-02	0,863	0,016	0,936	0,2181
**RPS13**	1,73E-01	4,80E-09	1,73E-01	2,33E-02	2,10E-01	4,14E-03	1,77E-01	1,93E-02	1,88E-01	1,20E-03	2,37E-01	2,47E-02	1,004	0,488	0,978	0,4108
