# Supplementary material for: A Versatile Strategy to Reduce UGA-Selenocysteine Recoding Efficiency of the Ribosome Using CRISPR-Cas9-Viral-Like-Particles Targeting Selenocysteine-tRNA[Ser]Sec Gene
Source: Cells. 2019 Jun 11;8(6):574. doi: 10.3390/cells8060574 (PMC6627462; doi:10.3390/cells8060574)
Supplement: Supplementary file 1 [file cells-08-00574-s001.zip › supplementary/Table S3.pdf]

Table S3. Values of Selenoprotein mRNA levels in response to the CRISPR-Cas9-VLP treatments and/or addition of selenium (100 nM) in Hek293 cell lines obtained by RT-qPCR. The geometrical mean of five housekeeping genes (Hpcb, Rps13, rRNA 18S, Hrpt and Gapdh) was calculated and used to normalize mRNA abundance.

**VLP**	**No**		**tRNA**		**EMX**		**No**		**tRNA**		**EMX**		**No VLP/tRNA VLP**		**Ctl/100 nMSe (No VLP)**	
**Selenium**	**Ctl**		**Ctl**		**Ctl**		**100 nM**		**100 nM**		**100 nM**					
	Ave	SD	Ave	SD	Ave	SD	Ave	SD	Ave	SD	Ave	SD	Fold change	p value	Fold change	p value
Selenoprotein genes																
**Gpx4**	2,70E-01	6,95E-02	1,85E-01	1,32E-03	3,97E-01	3,55E-02	3,47E-01	6,69E-02	2,65E-01	1,86E-02	3,53E-01	3,04E-02	1,459	0,1698	0,777	0,0076
**Gpx1**	1,18E-01	3,85E-03	7,37E-02	3,20E-03	1,20E-01	3,17E-03	1,02E-01	9,94E-03	6,49E-02	2,22E-03	9,97E-02	8,31E-03	1,602	0,0356	1,164	0,1687
**SELENOP**	8,17E-02	4,32E-03	5,78E-02	3,49E-04	7,81E-02	1,56E-02	8,11E-02	1,03E-03	6,46E-02	8,07E-04	8,30E-02	9,08E-03	1,414	0,0436	1,008	0,4476
**TXNRD1**	6,35E-02	2,10E-03	7,39E-02	2,68E-03	6,46E-02	1,65E-04	5,62E-02	1,99E-03	7,16E-02	3,48E-03	5,32E-02	2,67E-03	0,860	0,0127	1,129	0,0033
**SELENOI**	3,71E-02	1,94E-05	3,24E-02	3,23E-04	3,26E-02	3,07E-04	2,79E-02	1,97E-03	2,93E-02	2,87E-03	3,07E-02	9,84E-04	1,147	0,0144	1,331	0,0472
**SELENOT**	2,36E-02	1,16E-04	1,68E-02	1,68E-04	2,23E-02	6,63E-04	2,08E-02	1,12E-03	1,64E-02	6,22E-04	1,93E-02	7,14E-05	1,407	0,0017	1,139	0,0937
**SELENOK**	2,13E-02	3,21E-03	2,45E-02	6,18E-04	1,47E-02	3,20E-03	1,87E-02	4,67E-03	1,77E-02	5,63E-03	1,04E-02	2,17E-03	0,869	0,2224	1,138	0,3620
**TXNRD2**	1,65E-02	6,24E-10	1,42E-02	4,17E-03	1,82E-02	3,35E-03	1,49E-02	7,09E-03	1,44E-02	4,43E-03	1,51E-02	3,67E-03	1,166	0,2857	1,109	0,4007
**SELENOF**	5,96E-03	3,04E-04	3,81E-03	1,97E-03	5,24E-03	1,59E-04	5,05E-03	1,69E-04	5,13E-03	2,30E-03	4,86E-03	1,13E-03	1,565	0,2044	1,180	0,1123
**SELENOO**	3,20E-03	6,51E-05	2,54E-03	1,04E-03	4,50E-03	7,79E-05	3,74E-03	4,81E-05	4,28E-03	3,04E-04	2,68E-03	1,95E-03	1,259	0,2776	0,857	0,0072
**SELENOV**	1,46E-03	3,63E-04	6,76E-04	9,49E-04	1,31E-03	1,15E-04	1,03E-03	2,65E-04	1,13E-03	3,30E-04	1,07E-03	5,61E-05	2,158	0,1549	1,412	0,0517
**MSRB1**	1,40E-03	7,37E-04	2,07E-03	2,55E-05	2,03E-03	1,55E-04	1,89E-03	3,59E-04	2,01E-03	3,21E-05	1,57E-03	2,96E-04	0,679	0,2170	0,743	0,1601
**DIO2**	1,13E-03	2,05E-04	7,73E-04	2,57E-04	5,54E-04	1,53E-04	7,30E-04	2,18E-04	9,97E-04	7,75E-05	6,91E-04	8,47E-05	1,464	0,0326	1,551	0,2036
**SEPHS2**	7,62E-04	2,64E-05	1,14E-03	2,82E-04	9,85E-04	5,14E-04	8,94E-04	8,60E-05	1,39E-03	3,84E-04	2,03E-03	1,33E-03	0,667	0,1660	0,852	0,1719
**SELENOS**	4,40E-04	2,65E-04	5,61E-04	3,31E-04	5,05E-04	1,07E-04	8,10E-04	3,57E-05	9,33E-04	2,97E-04	1,02E-03	1,18E-04	0,783	0,4106	0,543	0,1316
**GPX3**	2,64E-04	7,30E-05	4,20E-04	2,09E-04	3,13E-04	7,87E-05	3,53E-04	7,07E-05	2,83E-04	8,63E-05	3,49E-04	5,46E-05	0,628	0,2881	0,747	0,0057
**SELENOH**	1,67E-04	1,90E-05	2,13E-04	5,97E-05	2,80E-04	9,69E-06	3,88E-04	2,86E-05	2,96E-04	2,70E-05	4,77E-04	1,47E-05	0,782	0,1770	0,430	0,0481
**SELENON**	1,53E-04	5,98E-05	1,34E-04	8,96E-05	3,13E-04	6,76E-05	3,24E-04	1,09E-07	3,41E-04	7,73E-05	3,63E-04	1,73E-04	1,148	0,2600	0,473	0,0773
**SELENOW**	1,29E-04	3,25E-05	1,50E-04	4,12E-05	1,86E-04	3,82E-05	4,57E-04	6,38E-05	2,41E-04	3,63E-05	4,47E-04	9,50E-05	0,864	0,0930	0,283	0,0215
**TXNRD3**	8,39E-05	2,64E-05	9,88E-05	4,63E-05	1,37E-04	6,64E-05	2,05E-04	9,13E-05	3,44E-04	9,68E-12	2,92E-04	9,24E-05	0,850	0,2415	0,410	0,1155
**SELENOM**	7,29E-05	2,66E-05	6,06E-05	2,05E-06	2,85E-05	1,70E-05	1,98E-04	8,83E-05	1,49E-04	1,30E-04	1,24E-04	2,31E-05	1,203	0,3260	0,368	0,1068
**DIO3**	1,38E-05	6,45E-06	9,48E-06	4,62E-07	9,86E-06	2,95E-06	9,71E-06	3,76E-07	1,17E-05	1,97E-06	9,25E-06	4,15E-06	1,460	0,2682	1,425	0,2565
Reference genes																
**GAPDH**	2,46E+01	3,89E-01	1,78E+01	1,63E-01	1,68E+01	6,96E+00	1,57E+01	4,78E-01	1,46E+01	5,74E-01	1,38E+01	4,44E-02	1,383	0,0074	1,573	0,0022
**18S**	1,79E+01	2,94E+00	1,62E+01	2,44E-01	1,65E+01	7,13E-01	1,77E+01	4,98E-03	1,68E+01	3,40E+00	1,56E+01	3,15E-01	1,104	0,2699	1,013	0,4653
**HPRT**	2,26E-01	7,54E-03	2,06E-01	6,01E-03	2,19E-01	5,16E-03	2,01E-01	2,96E-02	2,10E-01	2,79E-02	1,99E-01	1,53E-02	1,097	0,0171	1,123	0,2589
**HSPCB**	1,99E-01	7,21E-03	2,59E-01	4,13E-02	2,42E-01	1,16E-01	3,15E-01	4,65E-02	3,57E-01	5,52E-02	3,04E-01	1,81E-02	0,766	0,1639	0,630	0,1003
**RPS13**	5,04E-02	1,07E-03	6,47E-02	7,36E-03	6,81E-02	5,44E-03	5,69E-02	1,97E-02	5,44E-02	1,82E-02	3,86E-02	5,37E-02	0,779	0,0962	0,886	0,3545
